# Gradient Organization of Space, Time, and Numbers in the Brain: A Meta-analysis of Neuroimaging Studies

**DOI:** 10.1007/s11065-023-09609-z

**Published:** 2023-08-18

**Authors:** Giorgia Cona, Martin Wiener, Francesco Allegrini, Cristina Scarpazza

**Affiliations:** 1 Department of General Psychology, University of Padua, Via Venezia 8, 35131 Padua, Italy; 2 Padova Neuroscience Center, University of Padua, Padua, Italy; 3 Department of Neuroscience, University of Padua, Padua, Italy; 4 Department of Psychology, George Mason University, Fairfax, VA USA; 5 IRCSS San Camillo Hospital, Venice, Italy

**Keywords:** Meta-analysis, Magnitudes, Gradients, Neuroimaging, Activation likelihood estimation, Spatial, Numbers, Time

## Abstract

In this study, we ran a meta-analysis of neuroimaging studies to pinpoint the neural regions that are commonly activated across space, time, and numerosity, and we tested the existence of gradient transitions among these magnitude representations in the brain. Following PRISMA guidelines, we included in the meta-analysis 112 experiments (for space domain), 114 experiments (time domain), and 115 experiments (numerosity domain), and we used the activation likelihood estimation method. We found a system of brain regions that was commonly recruited in all the three magnitudes, which included bilateral insula, the supplementary motor area (SMA), the right inferior frontal gyrus, and bilateral intraparietal sulci. Gradiental transitions between different magnitudes were found along all these regions but insulae, with space and numbers leading to gradients mainly over parietal regions (and SMA) whereas time and numbers mainly over frontal regions. These findings provide evidence for the GradiATOM theory (Gradient Theory of Magnitude), suggesting that spatial proximity given by overlapping activations and gradients is a key aspect for efficient interactions and integrations among magnitudes.

**Supplementary Information:**

The online version contains supplementary material available at 10.1007/s11065-023-09609-z.

## Introduction

Quantity processing is an intrinsic and essential ability in humans; it emerges already during infancy and is observed even in nonhuman species (Haun et al., 2010). In particular, quantities such as spatial information, temporal representations, and numerosity representations permeate our mental life. Given the ubiquitous role of such quantity representations, it is crucial to understand how basic quantitative processing emerges from brain activity and how different dimensions interact with each other in the brain. How are these dimensions mapped in the brain? To what extent do they share brain regions or are instead linked to distinct neural structures? The present questions are the starting point of the study, which involved a meta-analysis of neuroimaging studies that explored numerical, temporal, or spatial processing.

### Behavioral Evidence of Interactions Across Space, Numbers, and Time

Behavioral studies unveiled intricate and intimate connections between numbers, space, and time (see Mix & Cheng, 2012; Hawes et al., 2019; Bonato et al., 2012; Ishihara et al., 2008). In their review, Mix and Cheng showed a large body of evidence for robust connections between math and space (Mix & Cheng, 2012). Mathematics is indeed often conceptualized in terms of spatial relations. An example is the so-called mental number line: Humans represent numbers and their inter-relations along a mental number line wherein numbers are arranged in ascending order from left-to-right. A number of findings such as the line bisection effects (Calabria & Rossetti, 2005) or the SNARC effect (spatial-numerical association of response codes; Dehaene et al., 1993) bring empirical support to this view. In particular, in SNARC paradigms, individuals are faster to judge whether a number is odd or even when responding with the left hand for small numbers and with the right hand for the larger numbers. This effect reflects the automatic link of small numbers (e.g., 1, 2, 3) to the left part of space and larger numbers (e.g., 7, 8, 9) to the right part of space.

A substantial literature revealed a pattern of interference also with temporal representations, as revealed, for example, by the STEARC effect (Spatial Temporal Association of Response Codes (Bonato et al., 2012; Ishihara et al., 2008), which refers to tendency to represent the concepts of past vs future, or before vs after along a horizontal spatial framework (e.g., line or arrow…) (Ishihara et al., 2008; Santiago et al., 2010; Torralbo et al., 2006; Vallesi et al., 2014; Weger & Pratt, 2008).

Interestingly, even if there is clear evidence for interference between space, time, and numbers, there are mixed results in the direction or symmetry of the interference.

A line of research showed an asymmetrical pattern of interference. Namely, spatial and numerical information were shown to alter temporal judgments, but temporal information does not bias spatial or numerical decision in either children (space: Bottini & Casasanto, 2013; number: Droit-Volet et al., 2003) or adults (space: Merritt et al., 2010; number: Dormal et al., 2006). On the other hand, another body of studies did reveal a symmetry in the interference, showing, for example, that judging stimulus length is influenced by stimulus duration, and vice versa (e.g., Cai & Connell, 2015). A key element of the dissociation relates to potential uncertainty and compatibility in the processing of each quantity. For example, numbers and lengths are capable of increasing and decreasing, whereas temporal duration can only increase—the so-called arrow of time (Riemer, 2015). Indeed, when quantities dynamically accumulate, time influences space and number, but not vice versa (Lambrechts et al., 2013; Martin et al., 2017). Taking together and despite some inconsistency in the reported direction of the interference phenomenon, behavioral data across quantity representations however provided some insights into the plausibility of a common magnitude system.

### Common and Distinct Brain Activations Across Space, Numbers, and Time

Parallels in clinical and neural investigations also led to similar conclusions. The most prominent theory in this sense is the “A Theory of Magnitude” (ATOM) by Walsh (2003), positing that time, space, and numbers are coded by a common magnitude system. According to the ATOM theory, there would be evolutionary reasons underlying this shared single system mainly located in the parietal cortex, which would indeed facilitate sensorimotor transformations and actions (Bueti & Walsh, 2009; Walsh, 2003).

A number of studies gave support to the existence of shared processing mechanisms between time, space, and numerosity (Cai & Connell, 2015; Schwiedrzik et al., 2016; Skagerlund et al., 2016; Srinivasan & Carey, 2010), showing overlapping brain activations between space and numbers (e.g., Hawes et al., 2019; Hubbard et al., 2005), numbers and time (Dormal et al., 2012b; Hayashi et al., 2013, 2015), and space and time (Cona et al., 2021), not only in parietal regions, but also in other regions such as frontal and insular regions.

More specifically, Hawes et al. (2019) conducted a meta-analysis of brain activations associated with mental rotation, basic symbolic number processing, and arithmetic, and found that all these cognitive processes share bilateral activations in parietal regions in and around the intraparietal sulcus (IPS) (see also Dormal & Pesenti, 2009; Dormal et al., 2012a). The authors interpreted this result adopting the “neuronal re-cycling hypothesis” (Dehaene & Cohen, 2007), according to which numbers may recruit or “re-use” that part of the brain devoted to spatial and sensorimotor operations. This more ancient and evolutionarily adaptive spatial system was originally used in the service of the external environment, in order to interact with objects, tools, and locations in space (Dehaene et al., 2003; Johnson-Frey, 2004; Lakoff & Núñez, 2000). In other words, “we may recycle the brain’s spatial prowess to navigate the ﻿abstract mathematical world” (Marghetis et al., 2014, p. 1580).

In addition to the parietal regions, studies have shown that numerical, mathematical, and visual-spatial processes activate the frontal lobes (Desco et al., 2011; Matejko & Ansari, 2015; O’Boyle et al., 2005; Zacks, 2008). Furthermore, a meta-analysis by Hawes et al. (2019) demonstrated consistent overlapping activations between space and numbers in frontal regions, particularly the left middle frontal gyrus. It is however less clear the functional meaning of frontal activations. Given the well-established role of prefrontal cortex in top-down control processes (Owen et al., 2005) and the evidence of an increased activation of dorsolateral prefrontal cortex (DLPFC) as a function of task difficulty (e.g., Kroger et al., 2002), the overlapped activation found in left middle frontal gyrus may reflect processes implied in the mental manipulation of information.

Common brain activations were also found between time and numbers, with a consistent involvement of a large fronto-parietal network that was lateralized over right hemisphere and that included the IPS, and frontal areas in the precentral, middle, and superior frontal gyri (e.g., Dormal et al., 2012b; Hayashi et al., 2013). Dormal et al. (2012b) suggested that the right IPS represents a common magnitude process for both time and numbers, likely reflecting the encoding and accumulation of information, whereas the right frontal regions are more involved in working-memory storage and decision-making processes.

Concerning the regions shared between time and space processing, a very recent meta-analysis (Cona et al., 2021) unveiled that the IPS, bilateral insula, the pre-supplementary motor area (pre-SMA), and the right frontal operculum are commonly recruited in both the two quantities processing. Notably, this study identified spatial gradients in some of the shared regions, along which spatial and temporal representations are mapped and organized in the brain (Cona et al., 2021). More specifically, frontal and parietal regions showed a dorsal–ventral gradient: Space is mediated by dorsal frontal and parietal regions, whereas time processing recruits ventral frontal and parietal regions. The SMA showed an anterior–posterior gradient, with space being associated with more anterior regions (i.e., pre-SMA) and time with more posterior regions (SMA-proper). Based on these findings, the GradiATOM view has been developed, which can be conceptualized as an extension of the ATOM by Walsh (2003). According to the GradiATOM view, the spatial proximity given by gradient configuration would ensure an efficient interplay between space and time magnitudes into a coherent representation of the external world that would then be adopted to prepare the appropriate action.

Interestingly, a similar network of regions was found to be commonly activated in three different magnitude processing tasks involving numerical, temporal, and spatial material, respectively (Skagerlund et al., 2016). These regions may thus represent the best candidate to form the core neural system for magnitude processing.

Together with overlapping activations, an increasing body of evidence revealed striking dissociations in how each magnitude is processed (e.g., Harvey et al., 2015; see Hamamouche & Cordes, 2019, for a review). For example, subcortical areas (e.g., globus pallidum, putamen caudate nucleus) have been identified as the primary locus of time processing (Nani et al., 2019; Teghil et al., 2019; Wiener et al., 2010), while they have no a crucial role in space and numerosity processing. Also, while there is a general consensus that parietal cortex, and right IPS in particular, is a cross-domain structure, commonly activated in the three domains, it was however shown that the left IPS is involved during numbers processing only and a right lateralized occipitoparietal network in spatial processing (Dormal & Pesenti, 2009; Hawes et al., 2019). The three magnitudes are thus also processed by distinct and specific neural structures.

### The Present Study

Despite numerous behavioral and neural commonalities shown between space and time (e.g., Cona et al., 2021), numbers and space (Hawes et al., 2019), and numbers and time (Dormal et al., 2012b), and the increasing body of evidence supporting the ATOM theory (﻿Bueti & Walsh, 2009; Walsh, 2003), no study has clearly pinpointed the “core network of magnitude” so far. Indeed, there is no a study that, using a meta-analytical approach able to wash out the idiosyncrasies of the specific paradigm and stimuli, explored neural commonalities among space, time, and numbers together. The first aim of the present study was thus to fill this gap by carrying out a systematic ALE (activation likelihood estimation) meta-analysis on brain regions associated with spatial, temporal, and numerical cognition (aim 1a) in order to delineate what are the regions that are commonly and consistently activated across the three magnitudes (aim 1b), regardless of the specific task and type of stimuli.

The second aim of the present study was to explore not only where the domains overlap in the brain, but also the extent of such overlap. We still have not a good understanding of where or how space, time, and numbers are related with each other in the brain; therefore, this is the first study to quantitatively describe the degree to which the three domains share neural activations in the brain regions that are identified as cross-domain structures.

Third, we better explored spatial organization among the domains, focusing on testing the existence of a gradient transition of time, space, and number representation in the brain. The idea of brain gradients is relatively new but is achieving increasing attention in literature (Huntenburg et al., 2018). A gradient is conceptualized as an axis of variance in structural and functional neural characteristics, along which brain areas are located in a spatially contiguous order; areas that resemble each other in relation to those characteristics lie in closer positions along the gradient. A previous study brought the first evidence for gradients that separate time from space processing in frontal and parietal regions (Cona et al., 2021). In the present study, we analyzed whether and how numerical processing is organized and represented along the same gradients that distinguish space-related and time-related neural processes.

By addressing the three goals, we sought out to provide an exhaustive delineation of the organization and reciprocal relationships of the time, space, and numbers in the brain.

## Material and Methods

### Studies Selection and Inclusion Criteria

The procedure for study selection for studies involving space and time processing is described in detail elsewhere (Cona et al., 2021). In the current paper, the very same procedure was applied to select eligible studies on number processing.

For number processing, an in-depth search was conducted up to May 2020 on PubMed and MEDLINE databases, using different combinations of the following search terms: “addition,” “arithmetic,” “counting,” “division,” “mathematic,” “number,” “SNARC,” “math,” “multiplication,” “arabic,” “calculation,” “comparison,” “magnitude,” “subtraction,” “calculation,” “numerosity,” “numerical,” “fMRI,” and “PET.” The full text of 193 possible eligible papers was accessed for eligibility. These papers were identified through database search and by tracing the references from identified papers, review articles, and previous meta-analyses (Arsalidou & Taylor, 2011; Hawes et al., 2019; Sokolowski et al., 2017).

In this study, studies were included if they met the following criteria:

(i) Studies using functional magnetic resonance imaging (fMRI) or positron emission tomography (PET) (i.e. EEG studies were excluded).
(ii) Studies analyzing the data using univariate approach that revealed localized increased activation for the processing of numerical material (i.e., studies using machine learning and multivoxel pattern analysis were excluded; studies analyzing the data using functional connectivity or related techniques have been excluded).
(iii) Studies performed a whole brain analysis (i.e., articles that performed only region of interest (ROI) or small volume correction (SVM) analysis have been excluded as they are known to create a bias (Kriegeskorte et al., 2009) and to impact on meta-analyses results (Gentili et al., 2019).
(iv) Studies that are peer-reviewed articles reporting novel data on spatial/temporal/numerical processing in healthy individuals (i.e., studies on pathological population have been excluded or only the data of the control group has been included if available).
(v) Studies that report a clear higher activation during spatial/temporal/numerical processing compared with a control condition (i.e., decreased activations were not included, as well as studies not using a control condition).
(vi) Studies including at least 5 participants.
(vii) Studies that report results in a standardized coordinate space (e.g., Talairach & Tournoux, 1988), or Montreal Neurologic Institute (MNI).

The database of the three domains with the studies and the resulting coordinates can be found on the following OSF folder: https://osf.io/qwrg4/?view_only=66b24545c5ec4a9a9010fc2fd624b592. Please cite the present study if you use them.

### Systematic Review

The literature screening and final selection have been performed according to the PRISMA guidelines (Liberati et al., 2009; Moher et al., 2009; Page et al., 2020). Applying the PRISMA procedure and the inclusion criteria, a total of 110 original articles were found eligible to be included in the systematic review on number processing (Supplementary Information A), 110 for space processing and 110 for time processing (see Cona et al., 2021).

Therefore, a total of 115 experiments (numerosity domain), 112 experiments (for space domain), and 114 experiments (time domain) have been included in the analysis (some articles contained multiple contrasts eligible for the analysis).

One author and one student extracted and checked the data independently. One author double-checked random data and double-checked data in case of discordance between the first two extractions, while another author was consulted in case of discordance or uncertainty. A dataset was created with the following features of each study: the number of subjects, the specific task used, the contrast performed, the coordinate system, the coordinate localization (brain regions), the *p* value criteria (corrected, uncorrected), and the associated statistic (*t* value, z score).

In order to avoid dependency across experiment maps that might negatively impact on the validity of the meta-analysis results, for each included study, only the contrast that most strongly reflected the process that the current meta-analysis aimed to investigate has been selected, in line with the recent meta-analysis guidelines (Muller et al., 2018). The full list of studies included for the numerical processing domain can be seen in Supplementary Information B file. The list of studies for space and time domains is the same as that of Cona et al. (2021).

### ALE Meta-analysis

The current study followed the most recent guidelines for the meta-analysis (Muller et al., 2018; Supplementary Information C). Talairach coordinates were reported into MNI space before performing the meta-analysis using a linear transformation (Laird et al., 2010; Lancaster et al., 2007). For a quantitative assessment of inter study spatial convergence, the activation likelihood estimation (ALE) method (Eickhoff et al., 2009; Laird et al., 2005; Turkeltaub et al., 2002) has been applied. The peaks of enhanced activation during spatial, temporal, or numerical processing compared to the control condition were used to generate an ALE map, using the revised ALE algorithm (Turkeltaub et al., 2012) running under Ginger ALE software (http://brainmap.org/ale/) version 3.0.2. This approach aims to identify areas with a spatial (within the brain) convergence of reported coordinates across experiments that is higher than expected from a random distribution of foci. Briefly, this algorithm treats activated foci of brain regions as three-dimensional Gaussian probability distributions centered at the given coordinates (Eickhoff et al., 2009; Laird et al., 2005). The algorithm incorporates the size of the probability distributions by considering the sample size of each study. Moreover, the algorithm tests the above chance clustering between contrasts rather than the above-chance clustering between foci, thus applying the random-effect rather than the fixed-effect inference. Inference is then sought regarding regions where the likelihood of activation being reported in a particular set of experiments is higher than expected by chance, i.e., where there is a non-random spatial convergence across the brain. For further details on the ALE method, please refer to the original publications (Eickhoff et al., 2012; Turkeltaub et al., 2012). To investigate the neural activations associated with processing of spatial, temporal, or numerical information, ALE meta-analyses were run. Statistical ALE maps were thresholded using cluster level FWE correction at *p* < 0.05 (cluster-forming threshold at voxel-level *p* < 0.001) (Eickhoff et al., 2016) in line with the recent guidelines for coordinate-based meta-analysis (Muller et al., 2018).

Furthermore, as we were interested in understanding the brain regions specifically activated for number as compared with time and space, and vice versa, pairwise discriminability (i.e., subtraction) analysis was run between the ALE maps of number, space, and time. This procedure allows one to test if two sets of foci (i.e., cognitive function A vs cognitive function B) statistically differ in spatial convergence. To perform the discriminability analysis, the experiments contributing to either analysis (i.e., cognitive function A and cognitive function B) were pooled together and then, recursively for 5000 permutations, randomly divided into two groups of the same size as the original sets of data (Eickhoff et al., 2012). An empirical null distribution of ALE-score differences between the two conditions was created subtracting, for each of the 5000 permutations, the voxelwise ALE scores of these two randomly assembled sets of foci from one another. The true results were then compared with the null distribution. Based on this permutation procedure, the map of true differences was then thresholded using a corrected *p* < 0.05 and an extent threshold of 100 voxels was applied to eliminate minor, presumably incidental, findings.

To simplify interpretation of ALE contrast images, they are converted to Z scores to show their significance instead of a direct ALE subtraction. This discriminability analysis yielded three different outputs: brain regions that are specifically activated for cognitive function A as compared to cognitive function B (A > B); brain regions that are specifically activated for cognitive function B as compared to cognitive function A (B > A) (aim 1a); and brain regions that are similarly activated by the two domains (conjunction analysis between cognitive functions A and B). Furthermore, the percentage of overlap between numbers and space, numbers and time, and time and space was calculated (aim 2).

Finally, an overall conjunction analysis was run considering the three cognitive domains (i.e. numbers, space, and time) at the same time, to test the possible presence of common activations during the processing of numerical, spatial, and temporal information (aim 1b).

### Gradients Identification and Stability

In order to identify gradients of activation likelihood (aim 3), we compared ALE maps in a manner similar to a previous report (Cona et al., 2021). Specifically, thresholded ALE maps were compared for each of the magnitudes (time, space, number) by inverting the values for one and adding it to the other. For example, in the time-number comparison, the gradient map was calculated as ALE_gradient_ = ALE_time_ + (− ALE_number_), where positive and negative ALE values would indicate greater likelihood for time and number, respectively. Importantly, zero values would represent points of overlap, and gradients would be located in regions where ALE values spanned from positive to negative values (or vice versa) in a continuum.

Once gradient maps were generated, the reliability and stability of observed gradients were also determined as done previously (Cona et al., 2021). First, the reliability of gradients was measured by generating a null distribution for each gradient map. This was done by taking the coordinates for each meta-analysis, randomly scattering them across the brain, and then generating new, unthresholded ALE maps, which were combined into gradient maps as described above. This process was repeated 1000 times to generate a distribution representing gradients that could have arisen by chance. For stability of gradients, we again took the coordinates for each meta-analysis, selected a random subset of each (70%), generated un-thresholded ALE maps from them, and again combined them into gradient maps. This process was also repeated 1000 times to generate distributions representing the robustness of the gradients to removing a small number of studies (for a similar method of estimating gradient stability, see Vos de Wael et al., 2020).

## Results

All the results can be downloaded on the following OSF page: https://osf.io/qwrg4/?view_only=66b24545c5ec4a9a9010fc2fd624b592.

### Aim 1a: Identification of Brain Regions Consistently Activated During the Processing of Space, Time, and Numbers

**Fig. 1:**
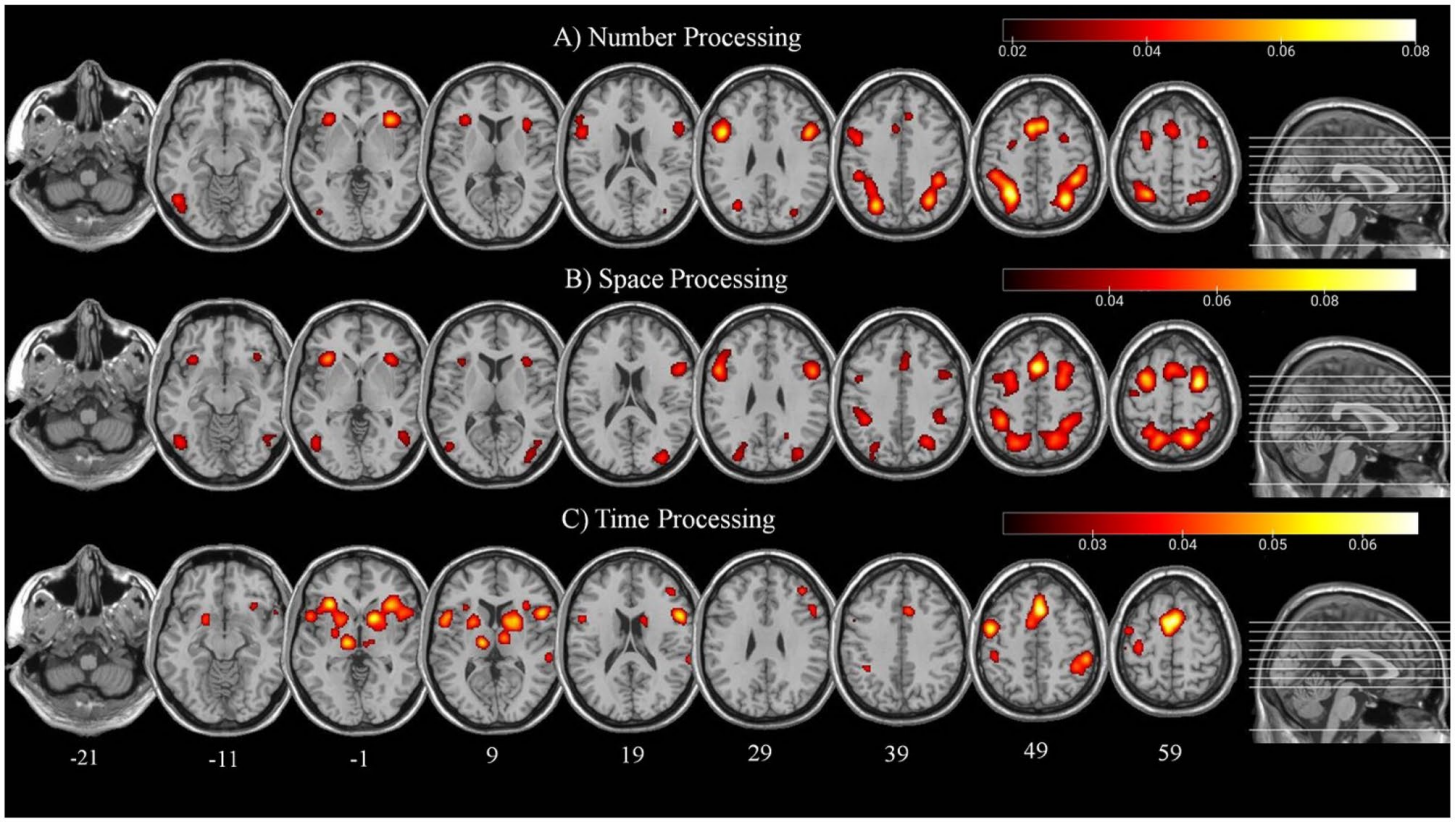
Significant convergent activations in studies on numbers processing (**A**), space processing (**B**), and time processing (**C**). Color bars indicate the ALE values for each voxel above the threshold (where yellow indicates the most significant ALE values). Numbers indicate the z coordinate for each section. Image created using MRIcro

The meta-analysis on studies showing greatest activation during a task involving the processing of numerical information rather than a control task was run on 1058 foci from 115 experiments, for a total of 1831 subjects. The minimum cluster size for the cluster to be considered statistically significant was 968 mm^3^. The results, reported in Table 1 and Fig. 1A, revealed regions of spatial convergence during tasks involving processing of numerical information in a network involving bilaterally the superior parietal lobule (SPL, BA 7), the inferior parietal lobule (IPL, BA 40, including the intraparietal sulcus – IPS), the precuneus (BA 7), the supplementary motor area (SMA, BA6,32), the anterior cingulated gyrus (ACC, BA32), the inferior frontal gyrus (IFG, BA9) and insula (BA13), the middle frontal gyrus (MFG, BA 6), and the fusiform gyrus on the left hemisphere only (BA 37,19).

**Table 1:** Significant activation likelihood clusters for the analysis of numbers processing

Cluster	Coordinates	Brain region	Brodmann area
1	− 28 − 56 50	SPL	7
	− 26 − 68 40	Precuneus	7
	− 44 − 38 44	IPL	40
2	32 − 64 46	SPL	7
	44 − 40 48	IPL	40
	40 − 42 42	IPL	40
	24 − 62 58	Precuneus	7
	32 − 76 28	Precuneus	31
3	4 16 50	SMA	6
	− 4 14 48	SMA	32
	8 28 36	ACC	32
4	− 46 10 28	IFG	9
	− 54 22 22	IFG	9
5	48 10 26	IFG/insula	9
6	− 26 − 4 54	MFG	6
7	− 48 − 62 − 12	Fusiform gyrus	37
	− 44 − 74 − 8	Fusiform gyrus	19
8	− 32 24 2	Insula	13
9	34 − 4 62	MFG	6

Coordinates are expressed in MNI space

*SPL* superior parietal lobule, *IPL* inferior parietal lobule, *SMA* supplementary motor area, *ACC* anterior cingulate cortex, *IFG* inferior frontal gyrus

Spatial convergence in studies showing greatest activation during a task involving the processing of spatial or temporal information is presented as in Cona et al. (2021). Briefly, the processing of spatial information (Fig. 1B) is associated with an increased activation of a bilateral and symmetrical network involving the dorsal parietal regions (precunei, SPL, and regions surrounding the intra-parietal sulcus (IPS)), MFG, and IFG including the frontal eye field (FEF), pre-SMA, and insulae. The processing of time information (Fig. 1C) is associated with an increased activation of a bilateral and quasi-symmetrical network involving the basal ganglia (globus pallidum, putamen, caudatum), thalamus, anterior insula, IFG, MFG, SMA (both pre-SMA and SMA proper), precentral gyrus, IPL (including IPS), middle temporal gyrus (MTG), and cerebellum.

**Fig. 2:**
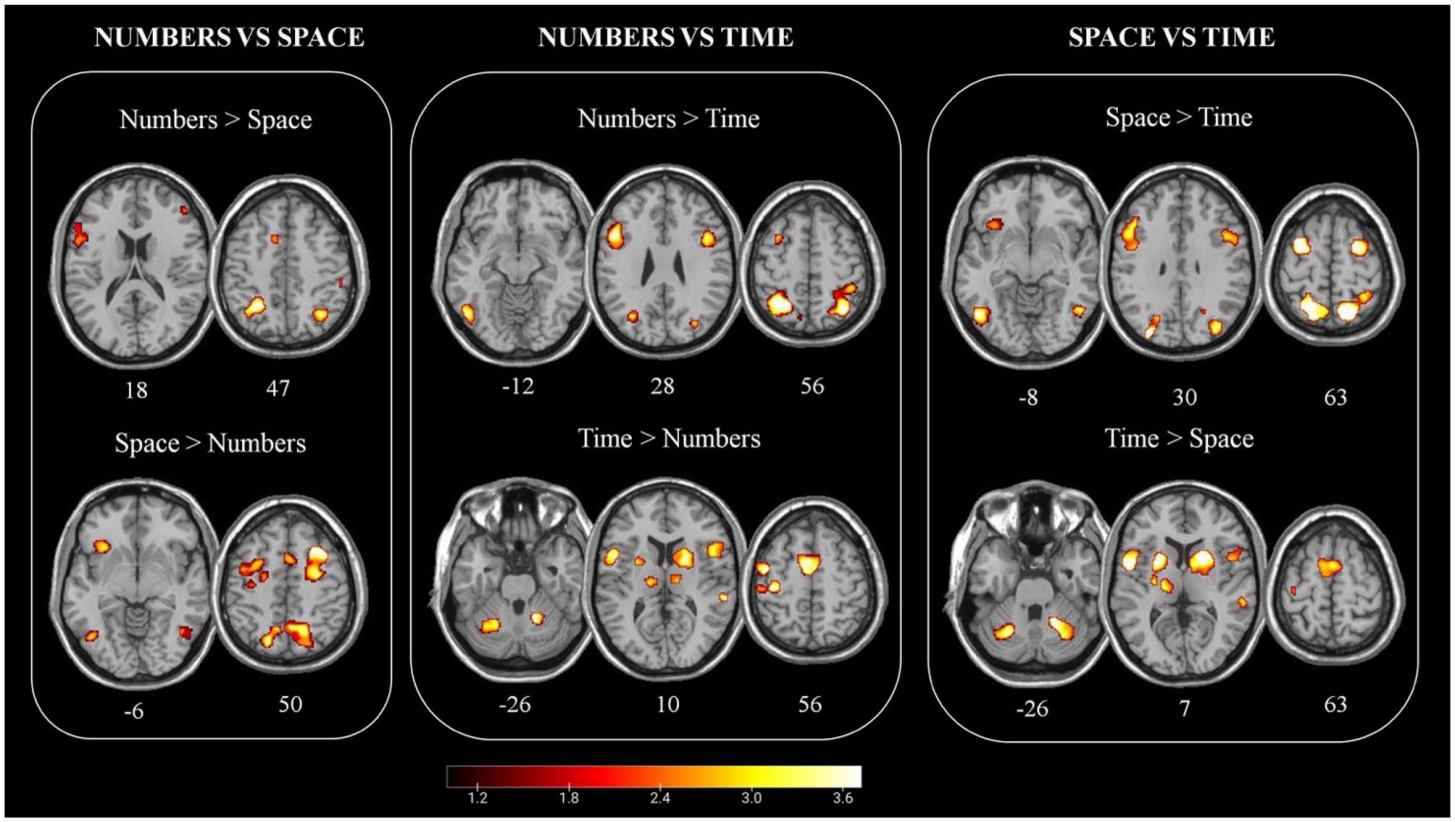
Pairwise direct comparison between numbers, space, and time. To simplify the interpretation of ALE contrast images, they are converted to z scores to show their significance instead of a direct ALE subtraction. Color bars indicate the z scores, where yellow indicates the higher z scores. Numbers indicate the z coordinate for each section. Image created using MRIcro

Pairwise contrast analyses (number vs space, number vs time, and space vs time) are represented in Fig. 2 and in Table 2.

**Table 2:** Significant activations comparing the three domains

	**Numbers vs Space**				**Numbers vs Time**				**Space vs Time**			
	***Numbers* > *Space***		***Space* > *Numbers***		***Numbers* > *Time***		***Time* > *Numbers***		***Space* > *Time***		***Time* > *Space***	
**Brain region**	**Coordinates**	**BA**	**Coordinates**	**BA**	**Coordinates**	**BA**	**Coordinates**	**BA**	**Coordinates**	**BA**	**Coordinates**	**BA**
SPL	− 26 − 53 47	7			− 28 − 60 46	7			− 27 − 45 50	7		
SPL									− 35 − 42 44	7		
SPL									− 54 − 30 40	7		
SPL			14 − 66 62	7	29 − 56 51	7			32 − 44 42	7		
SPL			25 − 51 70	7	24 − 64 60	7						
Precuneus	34 − 61 45	19	3 − 61 55	7	30 − 46 54	7			6 − 65 48	7		
Precuneus			22 − 76 50	7	26 − 44 52	7						
Precuneus			− 1 − 50 56	7	− 10 − 68 48	7			− 4 − 65 47	7		
IPL	36 − 48 36	40			42 − 42 56	40	60 − 34 47	40	36 − 36 38	40		
IPL	54 − 34 48	40									59 − 36 49	40
IPL			− 42 − 48 42	40	− 51 − 36 39	40	− 52 − 24 52	40				
IPL					− 46 − 40 46	40						
STG							56 16 − 12	22			60 − 32 4	22
STG							65 − 37 15	22				
MTG			50 − 63 − 1	37							60 − 36 2	22
MTG	− 32 − 62 34	39										
Angular	33 − 58 36	39			33 − 63 48	19						
IFG					44 10 28	9	54 20 6	44	46 9 26	9	49 5 4	44
IFG	− 58 22 18	45	− 32 22 − 11	47	− 48 13 26	9			− 48 14 27	6	− 52 7 8	44
IFG	− 40 12 26	9										
Insula							48 8 8	13				
Insula	− 30 28 8	13	− 42 22 2	13					− 32 20 − 14	13		
MCC	− 10 10 42	24										
PCC			22 − 58 22	31	36 − 74 24	31						
PCC			22 − 62 22	31								
SFG			− 30 2 68	6					− 22 − 12 46	6		
SFG									27 5 53	6		
MFG	− 50 10 38	9							− 29 2 60	6		
MFG	50 40 16	46	36 16 56	6					40 13 45	8		
MFG			− 22 0 62	6	− 54 28 20	46			− 42 24 30	9		
MeFG			− 18 − 8 50	6								
MeFG			16 8 56	6			2 26 44	8				
MeFG			12 12 60	6								
SMA			4 8 48	24			10 8 66	6			2 2 60	6
SMA			− 6 0 56	6	− 7 18 45	32	− 5 − 3 61	6	− 2 14 48	6	− 6 − 8 64	6
Precentral			49 4 46	6			46 10 4	44				
Precentral			48 2 42	6								
Precentral			− 37 − 2 54	6	− 42 0 30	6	− 51 5 5	44	− 26 − 14 46	6	− 53 − 6 51	6
Precentral									− 34 − 8 50	6	− 36 − 26 56	4
Postcentral			− 52 − 26 42	2			− 52 16 6	44				
Postcentral							− 38 − 26 57	40				
SOG			38 − 84 7	19								
SOG									− 24 − 68 26	39		
MOG			36 − 86 22	19					48 − 68 10	19		
MOG									26 − 86 20	19		
IOG			44 − 82 4	19								
IOG					− 40 − 76 − 2	19			− 47 − 67 − 5	19		
Fusiform					− 50 − 64 − 10	37						
Thalamus							15 − 19 − 1	-			10 − 18 0	50
Thalamus							− 14 − 13 − 3	-			− 12 − 18 2	50
Putamen							24 3 1	-			20 8 5	49
Putamen							− 24 4 − 5	-			− 16 6 10	49
Pallidum											24 − 2 − 4	51
Pallidum											− 18 − 3 1	51
Caudatum							10 10 8	-				
Cerebellum							17 − 54 21				19 − 55 − 25	-
Cerebellum							− 23 − 60 − 22	-			− 20 − 62 − 30	-

Coordinates are expressed in MNI space

*SPL* superior parietal lobule, *IPL* inferior parietal lobule, *STG* superior temporal gyrus, *MTG* middle temporal gyrus, *SMA* supplementary motor area, *MCC* middle cingulate cortex, *PCC* posterior cingulate cortex, *IFG* inferior frontal gyrus, *MFG* middle frontal gyrus, *MeFG* medial frontal gyrus, *SOG* superior occipital gyrus, *MOG* middle occipital gyrus, *IOG* inferior occipital gyrus

### Aim 1b: Identification of Brain Regions Commonly Activated During the Processing of Space, Time, and Numbers

Pairwise conjunction analyses revealed that the brain network of numbers and space processing overlap in a specific network including IPL, SPL, precuneus, SMA, medial cingulate cortex (MCC), precentral gyrus, IFG, insula, and MFG (Table 3). The overlap between the network of numbers and the network of time processing is less extended, involving IPL bilaterally, SMA, MCC, IFG bilaterally, left insula, and left precentral gyrus (Table 3). The overlap between space and time involves IPL bilaterally, pre-SMA, insula bilaterally, right IFG, and left precentral and postcentral gyrus (Table 3).

**Table 3:** Pairwise conjunction analyses and overall conjunction

	***Numbers ∩ space***		***Numbers ∩ time***		***Space ∩ time***		***Overall conjunction***	
**Brain region**	**Coordinates**	**BA**	**Coordinates**	**BA**	**Coordinates**	**BA**	**Coordinates**	**BA**
IPL	40 − 42 44	40	44 − 42 44	40	42 − 44 44	40	45 − 41 47	40
IPL			50 − 36 48	40				
Precuneus	28 − 66 42	7						
SPL	28 − 64 50	7						
SPL	32 − 54 53	7						
Precuneus	24 − 62 58	7						
Precuneus	32 − 76 28	31						
IPL	− 34 − 46 46	40	− 44 − 34 46	40	− 36 − 46 40	40	− 39 − 45 43	40
IPL	− 42 − 40 44	40	− 36 − 46 40	40				
Precuneus	− 26 − 68 46	7						
Precuneus	− 24 − 56 58	7						
Precuneus	− 26 − 74 30	31						
SMA	4 16 50	6	4 16 50	6	4 18 48	8	4 16 52	6
SMA	− 4 14 48	32			0 8 54	6		
Precentral Gyrus			− 48 2 42	6	− 42 0 50	6		
Postcentral Gyrus					− 44 − 34 46	40		
MCC	8 28 36	32	6 26 40	32				
IFG	− 48 12 28	9	− 52 8 18	44				
IFG	50 12 26	9	52 12 22	9	54 12 20	44	54 14 22	9
MFG	− 26 − 4 54	6						
Insula	− 32 24 0	13	− 32 24 2	13	− 30 24 − 2	13	− 30 23 0	13
Insula					36 22 0	13	37 23 0	13
MFG	34 − 4 62	6						

Coordinates are expressed in MNI space

*SPL* superior parietal lobule, *IPL* inferior parietal lobule, *SMA* supplementary motor area, *MCC* middle cingulate cortex, *IFG* inferior frontal gyrus, *MFG* middle frontal gyrus

**Fig. 3:**
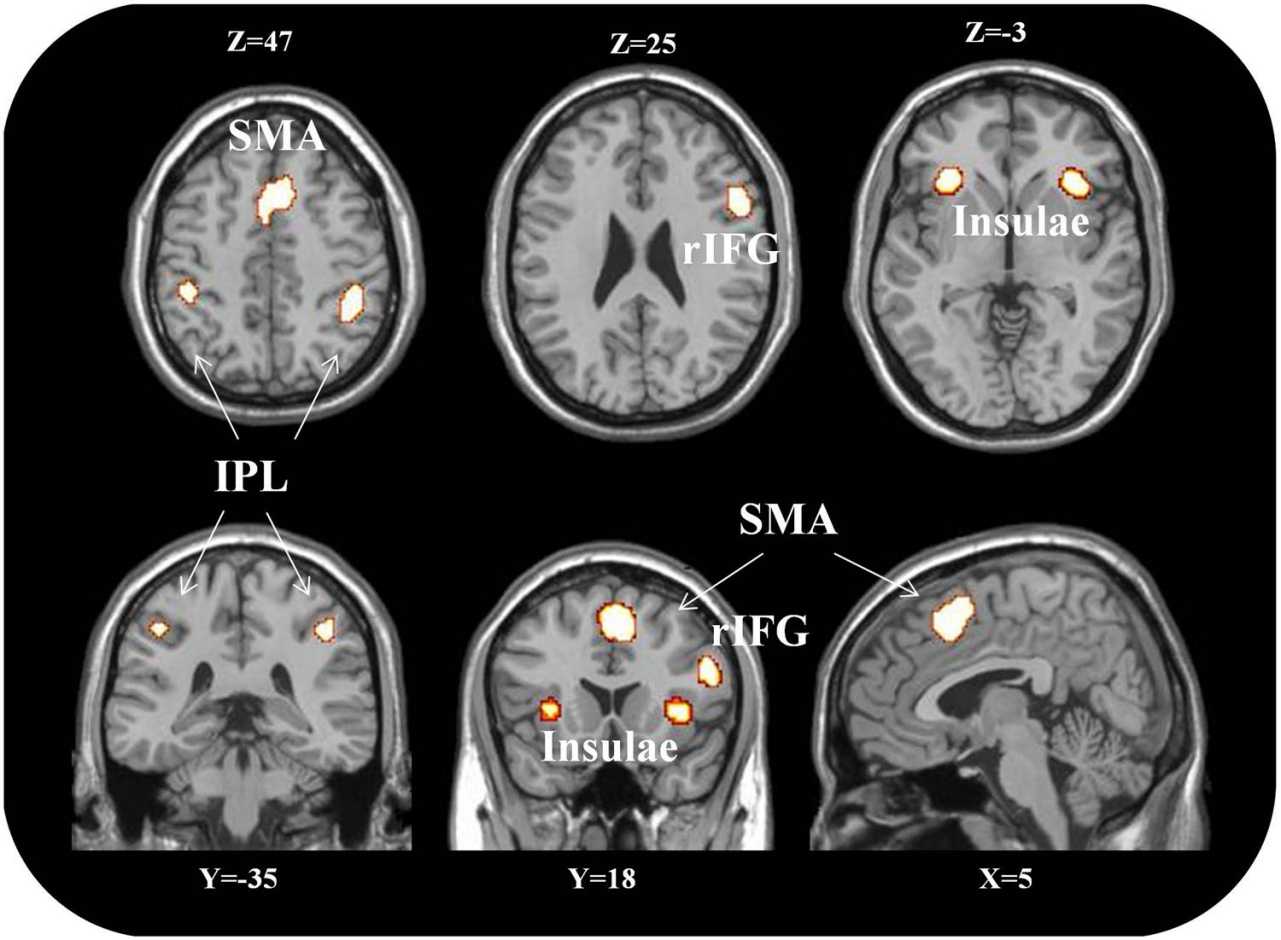
Regions of overlap between numbers, space and time. SMA, supplementary motor area; rIFG, right inferior frontal gyrus; IPL, inferior parietal lobule. For each section, the correspondent coordinate has been reported. Image created using MRIcro

The brain activations of the three cognitive functions overlap in the following brain regions: SMA/ACC (4, 16, 52), bilateral insula (− 30, 23,0 and 37, 23, 0), right IFG (54, 14, 22) and bilateral IPL (− 39, − 45, 43 and 45, − 41, 47). The results of this last conjunction analysis are visually represented in Fig. 3 (see also Table 3).

### Aim 2: Quantification of the Overlap Among the Three Cognitive Domains

Overall, the extent of the overlap between the networks of brain regions consistently activated during tasks involving numerical processing (numbers network) and spatial processing (space network) (37%) is the highest, while the overlap between the numbers network and time network was lowest (14%). The overlap between space and time networks appears to be similar to time and number (12%). This pattern is stable across each of the regions of interest (ROIs) emerging from the overlap between the three networks (see previous point). Percentages of overlap for each ROIs are presented in Table 4.

**Table 4:** Percentage of overlap between the cognitive domains

**ROIs**	**Number/space**	**Number/time**	**Space/time**
SMA	63%	46%	59%
R_IFG	62%	16%	20%
R_Insula	48%	38%	22%
L_Insula	29%	21%	19%
R_IPL	30%	9%	15%
L_IPL	39%	10%	18%

*ROIs* region of interest, *SMA* supplementary motor area, *L* left, *R* Right, *IFG* inferior frontal gyrus, *IPL* inferior parietal lobule

### Aim 3: Testing the Existence of Gradients

Notably, some of the regions of common activation represent the “intersection” of topographical gradients, along which the networks are mapped and organized in the brain. For each of the regions of common activation, we tested the presence of gradients along the anterior–posterior and dorsal–ventral axes and evaluated their reliability and stability.

#### Numbers vs Space

When comparing numbers and space domain, the gradients were found less defined, likely because of the high overlap between the two domains. No reliable gradients were found over insular regions, SMA and over rIFG.

On the other hand, over parietal regions space and numbers were more nicely represented along gradients. In both left and right IPL, numbers were linked to more ventral parietal regions whereas space was associated with more dorsal regions.

#### Numbers vs Time

**Fig. 4:**
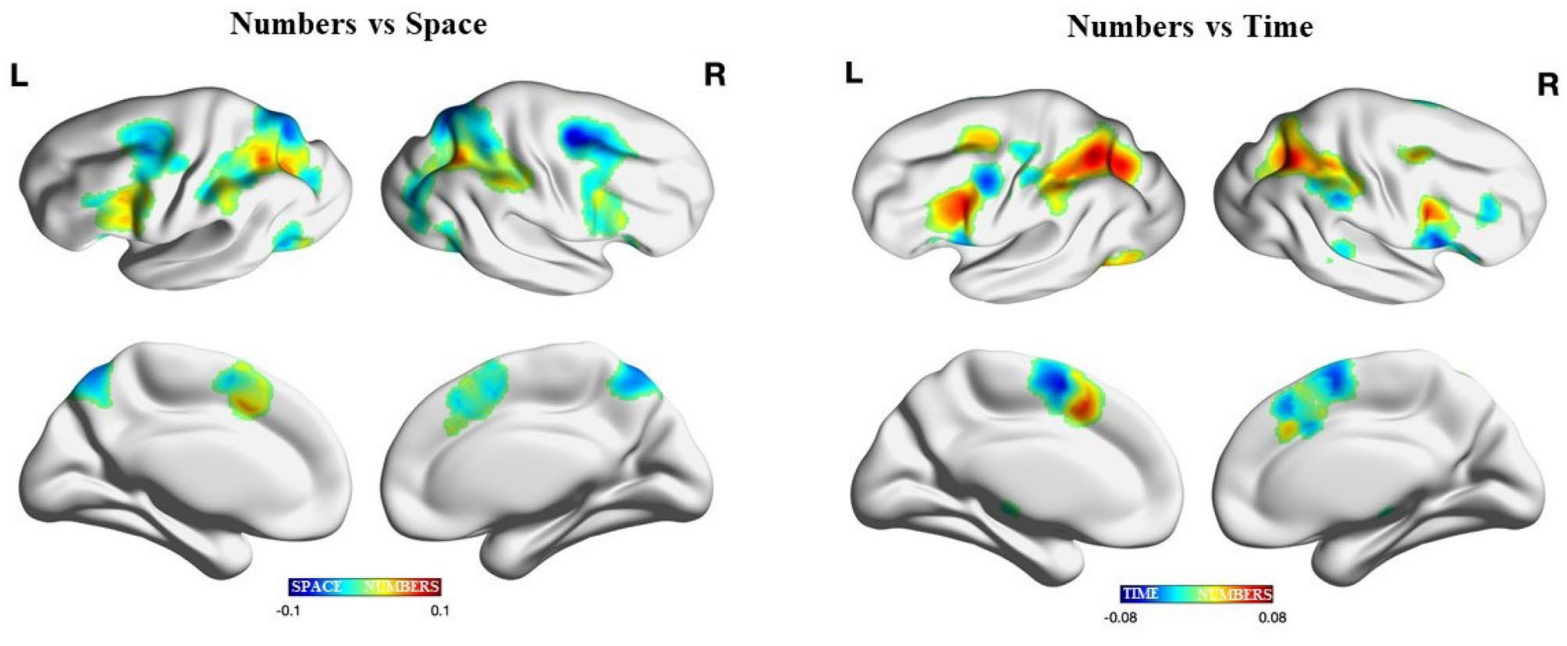
Gradient analysis. Surface visualization of the overlap between space and numbers (left panel) and time and numbers (right panel) meta-analyses. ALE values for space and time were set to negative scores and added to ALE values from the number meta-analysis, effectively subtracting one from the other

When comparing numbers and time domains, we found a well-defined anterior–posterior gradient over SMA regions, with time activating posterior regions while numbers being associated with more anterior regions (see Fig. 4). Such domains gave origin to another gradiental configuration over frontal regions, where an opposite pattern of gradients was found between left and right IFG. In the IFG, numbers were represented over more ventral regions, while time was instead represented over more dorsal regions. On the other hand, in the rIFG, numbers activated more dorsal frontal regions, while time activated more ventral regions. Over insular regions and parietal regions, no reliable gradients were instead observed.

#### Space vs Time

The pattern of gradients was analyzed and described in detail in a previous study (Cona et al., 2021) but, for the sake of completeness, is also reported very briefly here. We indeed found that the SMA exhibited an anterior (space)–posterior (time) gradient, while frontal and parietal regions showed a dorsal (space)–ventral (time) gradient.

**Fig. 5:**
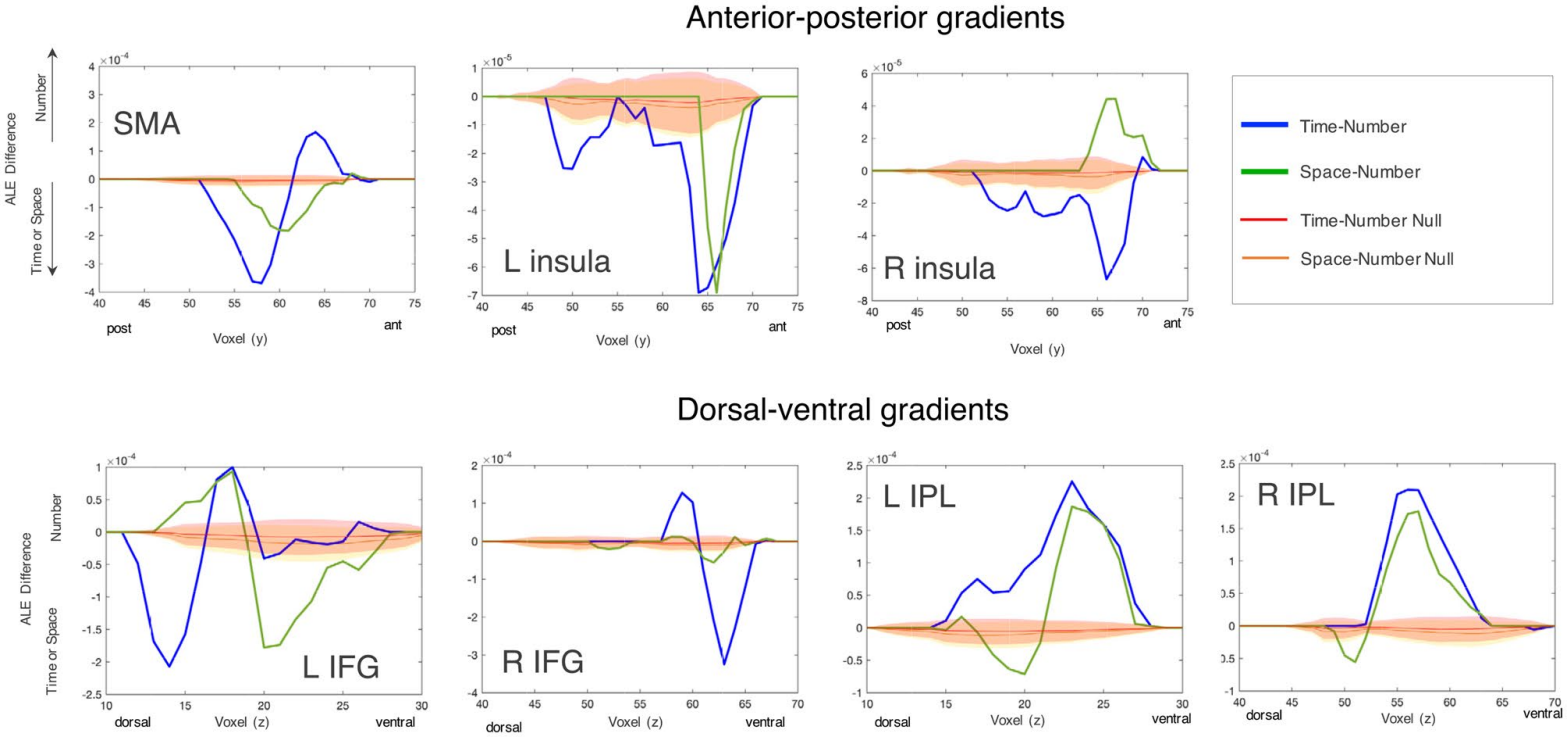
Gradient plots and stability. Plots of gradients across seven regions within the SMA, L/R IFG, L/R insula, and L/R IPL. Each plot displays the gradients observed for both time-number (blue trace) and space-number (green trace) contrasts, with greater number ALE scores always plotted as positive. Red and orange lines display the mean of the null distribution for time-number and space-number contrasts, respectively. Shaded regions represent the standard deviation of each distribution

For all gradients observed, we compared the observed gradients to null permutations for reliability and also compared to bootstrapped curves for reliability (Fig. 5). For all gradients observed, the shape and size exceeded that found in the null distribution.

## Discussion

Did evolution shape the human brain with a predisposition to represent magnitudes regardless of the specificity of the domains of knowledge? Where are domain-general and domain-specific neural activations located? In particular, how do space, time, and numbers interact with each other in terms of spatial activations? The present study tried to answer the following questions by using a quantitative meta-analytical approach.

### Core Network of Magnitude

The first aim of the study was to identify the brain regions shared among space, time, and numbers, highlighting in such a way the core network of magnitude. We found a set of areas to be conjointly activated across spatial, temporal, and numerosity tasks and that are located over SMA (extending to ACC regions), bilateral insulae, right IFG and bilateral IPL, around IPS sulci. As such, these regions are likely to be the best candidate for the core network of magnitude. This pattern of findings is coherent with one of the few recent studies that explored—within the same fMRI paradigm—the neural activations related to tasks involving processing of space, time, and numerosity (Skagerlund et al., 2016). Indeed, the conjunction analysis in the study by Skagerlund et al. (2016) identified the premotor cortex/SMA, insula, IFG and IPS, the key components of the magnitude network. However, they found that such a network was predominantly right-lateralized, while we found a quasi-symmetrical involvement of all these regions but IFG, which is mostly recruited over the right hemisphere. An explanation of such discrepancy might be the fact that this meta-analysis washed out possible idiosyncrasies related to the stimuli/tasks, which would lead to a different recruitment of the two hemispheres.

Looking closer at single regions, a shared activation of bilateral IPL regions in and around the IPS was found. This corroborates the notion that the IPS plays a critical role in the magnitude processing network and acts as a central hub responsible for the abstract representation beyond the specificity of the magnitude to code (Cona et al., 2021; Hawes et al., 2019; see Cohen Kadosh et al., 2008; Sokolowski et al., 2017). This is an important finding since, for more than two decades, the IPS has been conceptualized to house specific numerosity processes. In particular, one of the most prominent models in the numerical cognition field—the “Triple Code Model” (Dehaene & Cohen, 1997; Dehaene et al., 2003)—theorized that IPS supports the spatial and semantic representation and manipulation of numbers, as it is consistently involved during both symbolic and non-symbolic number tasks. Thus, the IPS has been theorized by this model to be the most plausible candidate for domain-specificity for numbers. Our results challenge this domain-specific view, showing instead that the IPS might play a more general role in magnitude processing (Leibovich et al., 2017; Walsh, 2003). This is coherent with the results from another recent meta-analysis by Hawes et al. (2019), which observed overlap between numbers, arithmetic processes, and mental rotation in and around the IPS, and proposed a general role of the IPS in judging magnitudes. Other studies including temporal tasks have also shown consistent activation of IPS regions (e.g., Walsh, 2003; see also previous meta-analyses: Cona & Scarpazza, 2019; Wiener et al., 2010), providing further evidence for a domain-general role. According to Walsh’s “a theory of magnitude” (ATOM), space, time, and quantity are alike in implying a goal-direction behavior; thus, they all represent items to be perceived or acted on (Walsh, 2003). Although more or less abstract, indeed, all magnitudes rely on neural mechanisms specialized for interacting with the physical world (e.g., see Anderson, 2010, 2014; Lakoff & Núñez, 2000; Marghetis et al., 2014). Recently, the IPS was shown to contain both topographic and chronotopic maps that are sensitive to specific features of spatial and temporal stimuli (Hagler & Sereno, 2006; Hayashi et al., 2015; Jerde & Curtis, 2013; Mackey et al., 2017; Teghil et al., 2019). The coexistence of both the maps within the same regions makes the IPS to be the best candidate for operations as transformation and integration of spatio-temporal information for action (Cona & Scarpazza, 2019).

The anterior cingulate cortex, bilateral anterior insula, and right frontal operculum are the key components of a network called salience network (Seeley et al., 2007) and cingulo-opercular control network (Dosenbach et al., 2008). These networks are involved in the transient identification of salient or relevant (either internal or external) stimuli from the continuous stream of stimuli and in marking such stimuli for additional processing in order to guide thoughts and behavior (Menon & Uddin, 2010; Seeley et al., 2007; Uddin, 2015). The salience network responds indeed to the degree of subjective salience, whether cognitive, emotional, or homeostatic (Goulden et al., 2014), and, based on such “salience,” this network drives the switching between the default mode network (DMN), which is active when the brain is not involved in a demanding cognitive task, and the central executive network, which is instead active when the brain is executing a task requiring attention. It is possible to apply the saliency hypothesis in the context of the present study, since the experiments included in the meta-analysis involved relevant or “salient” stimuli to process, irrespective of the nature of such stimuli (i.e., numerical, temporal or spatial).

Skagerlund et al. (2016), however, posited not to invoke the saliency hypothesis, and suggested a direct role for the insula (in particular) in magnitude processing. Interestingly, structural connectivity between insula and anterior IPS was shown in the study by Uddin et al. (2010), with IPS receiving inputs from visual cortices and sending this information via the dorsal visual stream to anterior insula (Uddin et al., 2010). In this way, the anterior insula would receive information from representations of magnitudes in the IPS (Harvey et al., 2013) and would mark events as quantity, spatial, or temporal units. Taking into account a recent neurocognitive model (Myers et al., 2017) for the role of cingulo-operculum network and applied in the field of spatial (Cona & Scarpazza, 2019) and temporal processing (Cona et al., 2021), the anterior insula and frontal operculum would serve to dynamically prioritize the representations of space/time/quantity units formed in the dorsal attention network, and more specifically in the IPS. The selected units would be combined in sequence in more integrated representation—likely by the pre-SMA—and then reformatted into an action-oriented format, as also proposed within the AtoM framework (Bueti & Walsh, 2009).

According to the “unified account” of the SMA, this region supports domain-general sequence operations in a variety of cognitive tasks (Cona et al., 2017; Leek et al., 2016; see Cona & Semenza, 2017, for a review). SMA regions, and pre-SMA in particular, play an essential role in the integration of sequential units into higher-order structural representations regardless of the kind of such units (spatial, motor, temporal, numerical, linguistic, and so forth) (Cona & Semenza, 2017). The evidence of gradients in the SMA regions, however, suggests that sub-regions of SMA are active preferentially for spatial/numerical stimuli (i.e., the anterior regions) or temporal stimuli (i.e., the posterior regions) (see paragraph below for a more detailed discussion of gradients).

Thus, along with a common network of regions, responsible for domain-general and operations shared by all the magnitudes, there is the activation of brain areas that are specifically involved in each magnitude and, over some cortical regions, gives rise to topographic gradients.

### Activation Overlaps and Gradients as Loci of Magnitudes Interaction

The analysis of overlap in each of the ROIs between the three magnitudes allowed us to have new insights on the extent to which distinct magnitudes share the same brain regions. In general, SMA has been revealed to be the brain region that shows the highest overlap across the distinct magnitudes (63% number/space; 46% number/time; 59% space/time). This would support its functional role as a supra-modal and domain-general hub that is critical for integrating and distributing higher-order information for the action (Cona & Semenza, 2017; Schwartze et al., 2012; Seghezzi & Zapparoli, 2020).

In particular, we found high activation overlap in the SMA between space and numbers, which gave no origin to a well-defined gradient. On the other hand, time and numbers—although sharing large part of the SMA—are also nicely represented along an antero-posterior SMA gradiental organization, with numbers activating more anterior regions (pre-SMA in particular) and time activating more posterior ones (SMA-proper). Likewise, a previous study (Cona et al., 2021) showed a gradient over SMA regions between space (anterior regions, pre-SMA) and time (posterior region, SMA-proper). Gradient anatomo-functional organization along the anterior–posterior axis of SMA has been demonstrated across several domains, both in humans and animals. In the timing domain, intrinsic functional organization of the SMA in gradients was found distinguishing temporal motor tasks, which activate SMA proper, from perceptual temporal tasks, which are associated with the pre-SMA activations (see also Wiener et al., 2010, 2011; Schwartze et al., 2012). Furthermore, temporal intervals are represented in different parts of SMA as a function of their duration, with pre-SMA and SMA-proper exhibiting preferential activations for short and long durations, respectively (Harvey et al., 2020; Protopapa et al., 2019).

Similar functional transitions were also shown in non-human models, and are paralleled by even more clear-cut rostro-caudal gradients in connectivity (Albertini et al., 2020). This study demonstrated in non-human primates a gradual functional transition from spatial representation of objects in anterior part of pre-SMA to visuomotor processing of self and other’s action, mapped in posterior parts. Also, this functional smooth transition in gradients is accompanied by an anterior–posterior transition in connectivity strength from lateral prefrontal cortex, and associative striatum, and anterior cingulate cortex (anteriorly), to dorso-ventral premotor cortices and putamen (posteriorly) (Albertini et al., 2020). The well-established architectural anatomo-functional homology between human and non-human primates’ SMA cortex (Nachev et al., 2008; Ruan et al., 2018) and the evidence of neurochemical and morphological smooth gradients in both species (Belmalih et al., 2007; Geyer et al., 1998) suggest that the gradients in connectivity profile observed in the study by Albertini et al. (2020) are likely paralleled by a similar organization principle of the human SMA connectivity as well. Further studies are needed to investigate possible magnitude-related gradual transitions in local connectional specificities in human brain.

In our study, spatial and numerical information are preferentially encoded by the anterior parts of SMA (the pre-SMA, in particular), while temporal information is instead associated with activation of posterior parts of SMA. According to the GradiATOM theory (Cona et al., 2021), gradients along SMA regions would enable an efficient integration of information derived from distinct domains to guide the appropriate action. The evidence of gradients and the high activation overlap among the three magnitudes support the crucial role of SMA as locus for the information interaction and integration for action since it is optimally placed for linking cognition to action (Nachev et al., 2008) and works as hub for motor intentionality-related processes (Zapparoli et al., 2018). Indeed, all these functional and structural properties make SMA to be “the most frequently activated region” in neuroimaging studies (Behrens et al., 2013).

Over parietal regions, we found a tight relationship between numbers and space. They indeed share high overlap in the activation of both left and right parietal regions, centered mainly around IPS, and they gave origin to a nicely defined gradient organization, with numbers activating ventral and space dorsal parietal areas. Together, these results are in line with the meta-analysis by Cantlon et al. (2009) and Cohen Kadosh et al. (2008), which investigated the spatial distribution of IPS activation across multiple magnitudes, finding that the IPS hosts both overlapping domain-specific and domain-general neural populations for numbers compared to non-numerical magnitudes (Cantlon et al., 2009; Cohen Kadosh et al., 2008). These meta-analyses, however, grounded their results on a qualitative method of visualizing data. Our meta-analysis used instead a quantitative meta-analytic tool, and provided statistical evidence for the existence of gradients around IPS.

On the other hand, gradiental transition from numbers to time-related activations is observable only over frontal regions, and more specifically over right inferior frontal gyrus and left medial frontal gyrus. Despite the role of frontal regions in magnitudes literature has been overlooked, recent meta-analytic studies found their crucial and consistent involvement in both numbers (e.g., Sokolowski et al., 2017) and time domains (Cona et al., 2021; Nani et al., 2019; Wiener et al., 2010) irrespective of the specific features of the stimulus to process, such as symbolic vs non-symbolic numerical quantities (Sokolowski et al., 2017), sub- vs supra-second temporal durations, or motor vs perceptual temporal tasks (Nani et al., 2019; Wiener et al., 2010). Therefore, such studies proposed a role related to higher order cognitive processes such as working memory, attentional selection, or inhibition (Nani et al., 2019; Sokolowski et al., 2017).

### Conclusions

The present study provides evidence for the existence of a core network of magnitude that is shared by space, time, and numbers, as proposed by the *ATOM* theory by Walsh (Bueti & Walsh, 2009; Walsh, 2003). This network encompasses SMA, bilateral insulae, right IFG and bilateral IPL, and around IPS sulci. Furthermore, gradiental transitions between different magnitudes were found along all these regions but insulae, with space and numbers leading to gradients mainly over parietal regions (and SMA) whereas time and numbers mainly over frontal regions. Space and time, instead, gave origin to gradients over both frontal and parietal regions. This pattern of finding is accurately explained by the *GradiATOM* theory (Cona et al., 2021), which posits that spatial proximity guaranteed by overlapping activations and gradients could be the key aspect for an efficient interaction and integration among different magnitudes. Further studies will be important to test this hypothesis using a within-subject design, wherein the time, space, and numbers magnitudes can be scientifically manipulated within the same experimental paradigm. Also, it will be interesting to explore whether gradients observed in brain activation parallel a similar gradiental organization in the functional or structural connectivity.

## Supplementary Information

Below is the link to the electronic supplementary material.

Supplementary file1 (DOCX 158 KB)

## Data Availability

Datasets of studies’ coordinates and results can be accessed on the following OSF page: https://osf.io/qwrg4/?view_only=66b24545c5ec4a9a9010fc2fd624b592. Please mention the present study if you use them. All the studies included in the meta-analysis are also cited in the Supplementary Materials.
