# Supplementary material for: Gradient Organization of Space, Time, and Numbers in the Brain: A Meta-analysis of Neuroimaging Studies
Source: Neuropsychol Rev. 2023 Aug 18;34(3):721–37. doi: 10.1007/s11065-023-09609-z (PMC11478975; doi:10.1007/s11065-023-09609-z)
Supplement: Supplementary file 1 — Supplementary file1 (DOCX 158 KB) [file 11065_2023_9609_MOESM1_ESM.docx]

**Supplementary Information A**

**Prisma Flow Diagram (for numbers domain)**

**Supplementary Information B**

**List of Included Studies for the “Numbers” meta-analysis**

(Abd Hamid et al., 2011; Andres et al., 2011; Andres et al., 2012; Ansari & Dhital., 2006; Ansari et al., 2005; Ansari et al., 2006a; Ansari et al., 2006b; Ansari et al., 2007; Attout et al., 2014; Audoin et al., 2005; Cantlon et al., 2006; Castelli et al., 2006; Chassy & Grodd, 2011; Chen et al., 2007; Chochon et al., 1999; Cohen Kadosh et al., 2005; Cohen Kadosh et al., 2007a; Cohen Kadosh et al., 2007b; Cohen Kadosh et al., 2011; Cowel et al., 2000; Damarla & Just, 2013; Daniels et al., 2003; De Pisapia et al., 2007; De Visscher et al., 2015; Dehaene et al., 1999; Delazer et al., 2003; Delazer et al., 2004; Demeyere et al., 2014; Dormal & Pesenti, 2009; Dormal et al., 2011; Eger et al., 2003; Eger et al., 2009; Fehr et al., 2007; Fehr et al., 2010; Fias et al., 2003; Fias et al., 2007; Franklin & Jonides 2008; Göbel et al., 2004; Goffin et al., 2019; Grabner et al., 2007; Grabner et al., 2009a; Grabner et al., 2009b; Grabner et al., 2012; Grodzinsky et al., 2020; Gruber et al., 2001; Gullick & Wolford, 2014; Hanakawa et al., 2003; Hayashi et al., 2000; Hayashi et al., 2013; Heidekum et al., 2019; Holloway et al., 2010; Holloway et al., 2013; Ischebeck et al., 2006; Ischebeck et al., 2007; Ischebeck et al., 2009; Jacob & Nieder, 2009; Jost et al., 2009; Kallai et al., 2012; Kansaku et al., 2007; Kaufmann et al., 2005; Kaufmann et al., 2008; Kawashima et al., 2004; Keller & Menon, 2009; Kong et al., 2005; Krueger et al., 2008; Kuo et al., 2008; Landrø et al.,2001.; Le Clec’H et al., 2000; Lee, 2000; Leroux et al., 2009; Liu et al., 2006; Lyons & Beilock, 2013; Menon et al., 2000; Menon et al., 2002; Molko et al., 2003; Ogata et al., 2011; Olm et al., 2014; Pesenti et al., 2000; Piazza et al., 2002; Piazza et al., 2006; Piazza et al.,2004; Piazza et al.,2007; Pinel et al., 1999; Pinel et al., 2001; Pinel et al., 2004; Prabhakaran et al., 2001; Prado et al., 2013; Rickard et al., 2000; Roggeman et al., 2011; Rosenberg-Lee, 2011; Sahan et al., 2018; Sammer et al., 2007; Santens et al., 2010; Simon et al., 2002; Stanescu-Cosson et al., 2000; Tan et al., 2007; Tenison et al., 2014; Tiberghien et al., 2019; van der Ven et al., 2016; Venkatraman et al., 2005; Venkatraman et al., 2006; Vogel et al., 2013; Vogel et al., 2017; Wood et al., 2008; Yang et al., 2017; Yi-Rong et al., 2011; Yusoff et al., 2014; Zago et al., 2001; Zago et al., 2008; Zhou et al., 2007)

Abd Hamid, A. I., Yusoff, A. N., Mukari, S. Z., & Mohamad, M. (2011). Brain Activation during Addition and Subtraction Tasks In-Noise and In-Quiet. *The Malaysian journal of medical sciences : MJMS*, *18*(2), 3–15.

Andres, M., Michaux, N., & Pesenti, M. (2012). Common substrate for mental arithmetic and finger representation in the parietal cortex. *NeuroImage*, *62*(3), 1520–1528. <https://doi.org/10.1016/j.neuroimage.2012.05.047>

Andres, M., Pelgrims, B., Michaux, N., Olivier, E., & Pesenti, M. (2011). Role of distinct parietal areas in arithmetic: an fMRI-guided TMS study. *NeuroImage*, *54*(4), 3048–3056. <https://doi.org/10.1016/j.neuroimage.2010.11.009>

Ansari, D., & Dhital, B. (2006). Age-related changes in the activation of the intraparietal sulcus during nonsymbolic magnitude processing: an event-related functional magnetic resonance imaging study. *Journal of cognitive neuroscience*, *18*(11), 1820–1828. <https://doi.org/10.1162/jocn.2006.18.11.1820>

Ansari, D., Dhital, B., & Siong, S. C. (2006a). Parametric effects of numerical distance on the intraparietal sulcus during passive viewing of rapid numerosity changes. *Brain research*, *1067*(1), 181–188. <https://doi.org/10.1016/j.brainres.2005.10.083>

Ansari, D., Fugelsang, J. A., Dhital, B., & Venkatraman, V. (2006b). Dissociating response conflict from numerical magnitude processing in the brain: an event-related fMRI study. *NeuroImage*, *32*(2), 799–805. <https://doi.org/10.1016/j.neuroimage.2006.04.184>

Ansari, D., Garcia, N., Lucas, E., Hamon, K., & Dhital, B. (2005). Neural correlates of symbolic number processing in children and adults. *Neuroreport*, *16*(16), 1769–1773. <https://doi.org/10.1097/01.wnr.0000183905.23396.f1>

Ansari, D., Lyons, I. M., van Eimeren, L., & Xu, F. (2007). Linking visual attention and number processing in the brain: the role of the temporo-parietal junction in small and large symbolic and nonsymbolic number comparison. *Journal of cognitive neuroscience*, *19*(11), 1845–1853. <https://doi.org/10.1162/jocn.2007.19.11.1845>

Attout, L., Fias, W., Salmon, E., & Majerus, S. (2014). Common neural substrates for ordinal representation in short-term memory, numerical and alphabetical cognition. *PloS one*, *9*(3), e92049. <https://doi.org/10.1371/journal.pone.0092049>

Audoin, B., Ibarrola, D., Au Duong, M. V., Pelletier, J., Confort-Gouny, S., Malikova, I., Ali-Chérif, A., Cozzone, P. J., & Ranjeva, J. P. (2005). Functional MRI study of PASAT in normal subjects. *Magma (New York, N.Y.)*, *18*(2), 96–102. <https://doi.org/10.1007/s10334-004-0098-9>

Cantlon, J. F., Brannon, E. M., Carter, E. J., & Pelphrey, K. A. (2006). Functional imaging of numerical processing in adults and 4-y-old children. *PLoS biology*, *4*(5), e125. <https://doi.org/10.1371/journal.pbio.0040125>

Castelli, F., Glaser, D. E., & Butterworth, B. (2006). Discrete and analogue quantity processing in the parietal lobe: a functional MRI study. *Proceedings of the National Academy of Sciences of the United States of America*, *103*(12), 4693–4698. <https://doi.org/10.1073/pnas.0600444103>

Chassy, P., & Grodd, W. (2012). Comparison of quantities: core and format-dependent regions as revealed by fMRI. *Cerebral cortex (New York, N.Y. : 1991)*, *22*(6), 1420–1430. <https://doi.org/10.1093/cercor/bhr219>

Chen, C., Zhou, X., Chen, C., Dong, Q., Zang, Y., Qiao, S., Yang, T., & Gong, Q. (2007). The neural basis of processing anomalous information. *Neuroreport*, *18*(8), 747–751. <https://doi.org/10.1097/WNR.0b013e3280ebb49b>

Chochon, F., Cohen, L., van de Moortele, P. F., & Dehaene, S. (1999). Differential contributions of the left and right inferior parietal lobules to number processing. *Journal of cognitive neuroscience*, *11*(6), 617–630. <https://doi.org/10.1162/089892999563689>

Cohen Kadosh, R., Bahrami, B., Walsh, V., Butterworth, B., Popescu, T., & Price, C. J. (2011). Specialization in the human brain: the case of numbers. *Frontiers in human neuroscience*, *5*, 62. https://doi.org/10.3389/fnhum.2011.00062

Cohen Kadosh, R., Cohen Kadosh, K., Kaas, A., Henik, A., & Goebel, R. (2007a). Notation-dependent and-independent representations of numbers in the parietal lobes. *Neuron*, *53*(2), 307-314.

Cohen Kadosh, R., Cohen Kadosh, K., Linden, D. E., Gevers, W., Berger, A., & Henik, A. (2007b). The brain locus of interaction between number and size: a combined functional magnetic resonance imaging and event-related potential study. *Journal of cognitive neuroscience*, *19*(6), 957–970. <https://doi.org/10.1162/jocn.2007.19.6.957>

Cohen Kadosh, R., Henik, A., Rubinsten, O., Mohr, H., Dori, H., van de Ven, V., Zorzi, M., Hendler, T., Goebel, R., & Linden, D. E. (2005). Are numbers special? The comparison systems of the human brain investigated by fMRI. *Neuropsychologia*, *43*(9), 1238–1248. <https://doi.org/10.1016/j.neuropsychologia.2004.12.017>

Cowell, S. F., Egan, G. F., Code, C., Harasty, J., & Watson, J. D. (2000). The functional neuroanatomy of simple calculation and number repetition: A parametric PET activation study. *NeuroImage*, *12*(5), 565–573. <https://doi.org/10.1006/nimg.2000.0640>

Damarla, S. R., & Just, M. A. (2013). Decoding the representation of numerical values from brain activation patterns. *Human brain mapping*, *34*(10), 2624–2634. <https://doi.org/10.1002/hbm.22087>

Daniels, C., Witt, K., Wolff, S., Jansen, O., & Deuschl, G. (2003). Rate dependency of the human cortical network subserving executive functions during generation of random number series--a functional magnetic resonance imaging study. *Neuroscience letters*, *345*(1), 25–28. <https://doi.org/10.1016/s0304-3940(03)00496-8>

De Pisapia, N., Slomski, J. A., & Braver, T. S. (2007). Functional specializations in lateral prefrontal cortex associated with the integration and segregation of information in working memory. *Cerebral cortex (New York, N.Y. : 1991)*, *17*(5), 993–1006. <https://doi.org/10.1093/cercor/bhl010>

De Visscher, A., Berens, S. C., Keidel, J. L., Noël, M. P., & Bird, C. M. (2015). The interference effect in arithmetic fact solving: An fMRI study. *NeuroImage*, *116*, 92–101. <https://doi.org/10.1016/j.neuroimage.2015.04.063>

Dehaene, S., Spelke, E., Pinel, P., Stanescu, R., & Tsivkin, S. (1999). Sources of mathematical thinking: behavioral and brain-imaging evidence. *Science (New York, N.Y.)*, *284*(5416), 970–974. <https://doi.org/10.1126/science.284.5416.970>

Delazer, M., Domahs, F., Bartha, L., Brenneis, C., Lochy, A., Trieb, T., & Benke, T. (2003). Learning complex arithmetic--an fMRI study. *Brain research. Cognitive brain research*, *18*(1), 76–88. <https://doi.org/10.1016/j.cogbrainres.2003.09.005>

Delazer, M., Domahs, F., Lochy, A., Bartha, L., Brenneis, C., & Trieb, T. (2004). The acquisition of arithmetic knowledge - an FMRI study. *Cortex; a journal devoted to the study of the nervous system and behavior*, *40*(1), 166–167. <https://doi.org/10.1016/s0010-9452(08)70936-5>

Demeyere, N., Rotshtein, P., & Humphreys, G. W. (2014). Common and dissociated mechanisms for estimating large and small dot arrays: value-specific fMRI adaptation. *Human brain mapping*, *35*(8), 3988–4001. <https://doi.org/10.1002/hbm.22453>

Dormal, V., & Pesenti, M. (2009). Common and specific contributions of the intraparietal sulci to numerosity and length processing. *Human brain mapping*, *30*(8), 2466–2476. <https://doi.org/10.1002/hbm.20677>

Dormal, V., Dormal, G., Joassin, F., & Pesenti, M. (2012). A common right fronto-parietal network for numerosity and duration processing: an fMRI study. *Human brain mapping*, *33*(6), 1490–1501. <https://doi.org/10.1002/hbm.21300>

Eger, E., Michel, V., Thirion, B., Amadon, A., Dehaene, S., & Kleinschmidt, A. (2009). Deciphering cortical number coding from human brain activity patterns. *Current biology : CB*, *19*(19), 1608–1615. <https://doi.org/10.1016/j.cub.2009.08.047>

Eger, E., Sterzer, P., Russ, M. O., Giraud, A. L., & Kleinschmidt, A. (2003). A supramodal number representation in human intraparietal cortex. *Neuron*, *37*(4), 719–725. <https://doi.org/10.1016/s0896-6273(03)00036-9>

Fehr, T., Code, C., & Herrmann, M. (2007). Common brain regions underlying different arithmetic operations as revealed by conjunct fMRI-BOLD activation. *Brain research*, *1172*, 93–102. <https://doi.org/10.1016/j.brainres.2007.07.043>

Fehr, T., Weber, J., Willmes, K., & Herrmann, M. (2010). Neural correlates in exceptional mental arithmetic--about the neural architecture of prodigious skills. *Neuropsychologia*, *48*(5), 1407–1416. <https://doi.org/10.1016/j.neuropsychologia.2010.01.007>

Fias, W., Lammertyn, J., Caessens, B., & Orban, G. A. (2007). Processing of abstract ordinal knowledge in the horizontal segment of the intraparietal sulcus. *The Journal of neuroscience : the official journal of the Society for Neuroscience*, *27*(33), 8952–8956. <https://doi.org/10.1523/JNEUROSCI.2076-07.2007>

Fias, W., Lammertyn, J., Reynvoet, B., Dupont, P., & Orban, G. A. (2003). Parietal representation of symbolic and nonsymbolic magnitude. *Journal of cognitive neuroscience*, *15*(1), 47–56. <https://doi.org/10.1162/089892903321107819>

Franklin, M. S., & Jonides, J. (2009). Order and magnitude share a common representation in parietal cortex. *Journal of cognitive neuroscience*, *21*(11), 2114–2120. <https://doi.org/10.1162/jocn.2008.21181>

Göbel, S. M., Johansen-Berg, H., Behrens, T., & Rushworth, M. F. (2004). Response-selection-related parietal activation during number comparison. *Journal of cognitive neuroscience*, *16*(9), 1536–1551. https://doi.org/10.1162/0898929042568442

Goffin, C., Vogel, S. E., Slipenkyj, M., & Ansari, D. (2020). A comes before B, like 1 comes before 2. Is the parietal cortex sensitive to ordinal relationships in both numbers and letters? An fMRI-adaptation study. *Human brain mapping*, *41*(6), 1591–1610. <https://doi.org/10.1002/hbm.24897>

Grabner, R. H., Ansari, D., Koschutnig, K., Reishofer, G., Ebner, F., & Neuper, C. (2009a). To retrieve or to calculate? Left angular gyrus mediates the retrieval of arithmetic facts during problem solving. *Neuropsychologia*, *47*(2), 604–608. <https://doi.org/10.1016/j.neuropsychologia.2008.10.013>

Grabner, R. H., Ansari, D., Reishofer, G., Stern, E., Ebner, F., & Neuper, C. (2007). Individual differences in mathematical competence predict parietal brain activation during mental calculation. *NeuroImage*, *38*(2), 346–356. <https://doi.org/10.1016/j.neuroimage.2007.07.041>

Grabner, R. H., Ischebeck, A., Reishofer, G., Koschutnig, K., Delazer, M., Ebner, F., & Neuper, C. (2009b). Fact learning in complex arithmetic and figural-spatial tasks: the role of the angular gyrus and its relation to mathematical competence. *Human brain mapping*, *30*(9), 2936–2952. <https://doi.org/10.1002/hbm.20720>

Grabner, R.H., Saalbach, H. and Eckstein, D. (2012), Language-Switching Costs in Bilingual Mathematics Learning. *Mind, Brain, and Education, 6*: 147-155. <https://doi.org/10.1111/j.1751-228X.2012.01150.x>

Grodzinsky, Y., Deschamps, I., Pieperhoff, P., Iannilli, F., Agmon, G., Loewenstein, Y., & Amunts, K. (2020). Logical negation mapped onto the brain. *Brain structure & function*, *225*(1), 19–31. <https://doi.org/10.1007/s00429-019-01975-w>

Gruber, O., Indefrey, P., Steinmetz, H., & Kleinschmidt, A. (2001). Dissociating neural correlates of cognitive components in mental calculation. *Cerebral cortex (New York, N.Y. : 1991)*, *11*(4), 350–359. <https://doi.org/10.1093/cercor/11.4.350>

Gullick, M. M., & Wolford, G. (2014). Brain systems involved in arithmetic with positive versus negative numbers. *Human brain mapping*, *35*(2), 539–551. <https://doi.org/10.1002/hbm.22201>

Hanakawa, T., Honda, M., Okada, T., Fukuyama, H., & Shibasaki, H. (2003). Differential activity in the premotor cortex subdivisions in humans during mental calculation and verbal rehearsal tasks: a functional magnetic resonance imaging study. *Neuroscience letters*, *347*(3), 199–201. <https://doi.org/10.1016/s0304-3940(03)00692-x>

Hayashi, M. J., Kanai, R., Tanabe, H. C., Yoshida, Y., Carlson, S., Walsh, V., & Sadato, N. (2013). Interaction of numerosity and time in prefrontal and parietal cortex. *The Journal of neuroscience : the official journal of the Society for Neuroscience*, *33*(3), 883–893. <https://doi.org/10.1523/JNEUROSCI.6257-11.2013>

Hayashi, N., Ishii, K., Kitagaki, H., & Kazui, H. (2000). Regional differences in cerebral blood flow during recitation of the multiplication table and actual calculation: a positron emission tomography study. *Journal of the neurological sciences*, *176*(2), 102–108. <https://doi.org/10.1016/s0022-510x(00)00323-3>

Heidekum, A. E., Grabner, R. H., De Smedt, B., De Visscher, A., & Vogel, S. E. (2019). Interference during the retrieval of arithmetic and lexico-semantic knowledge modulates similar brain regions: Evidence from functional magnetic resonance imaging (fMRI). *Cortex; a journal devoted to the study of the nervous system and behavior*, *120*, 375–393. <https://doi.org/10.1016/j.cortex.2019.06.007>

Holloway, I. D., Battista, C., Vogel, S. E., & Ansari, D. (2013). Semantic and perceptual processing of number symbols: evidence from a cross-linguistic fMRI adaptation study. *Journal of cognitive neuroscience*, *25*(3), 388–400. <https://doi.org/10.1162/jocn_a_00323>

Holloway, I. D., Price, G. R., & Ansari, D. (2010). Common and segregated neural pathways for the processing of symbolic and nonsymbolic numerical magnitude: an fMRI study. *NeuroImage*, *49*(1), 1006–1017. <https://doi.org/10.1016/j.neuroimage.2009.07.071>

Ischebeck, A., Zamarian, L., Egger, K., Schocke, M., & Delazer, M. (2007). Imaging early practice effects in arithmetic. *NeuroImage*, *36*(3), 993–1003. <https://doi.org/10.1016/j.neuroimage.2007.03.051>

Ischebeck, A., Zamarian, L., Schocke, M., & Delazer, M. (2009). Flexible transfer of knowledge in mental arithmetic--an fMRI study. *NeuroImage*, *44*(3), 1103–1112. <https://doi.org/10.1016/j.neuroimage.2008.10.025>

Ischebeck, A., Zamarian, L., Siedentopf, C., Koppelstätter, F., Benke, T., Felber, S., & Delazer, M. (2006). How specifically do we learn? Imaging the learning of multiplication and subtraction. *NeuroImage*, *30*(4), 1365–1375. <https://doi.org/10.1016/j.neuroimage.2005.11.016>

Jacob, S. N., & Nieder, A. (2009). Tuning to non-symbolic proportions in the human frontoparietal cortex. *The European journal of neuroscience*, *30*(7), 1432–1442. <https://doi.org/10.1111/j.1460-9568.2009.06932.x>

Jost, K., Khader, P., Burke, M., Bien, S., & Rösler, F. (2009). Dissociating the solution processes of small, large, and zero multiplications by means of fMRI. *NeuroImage*, *46*(1), 308–318. <https://doi.org/10.1016/j.neuroimage.2009.01.044>

Kallai, A. Y., Schunn, C. D., & Fiez, J. A. (2012). Mental arithmetic activates analogic representations of internally generated sums. *Neuropsychologia*, *50*(10), 2397–2407. <https://doi.org/10.1016/j.neuropsychologia.2012.06.009>

Kansaku, K., Carver, B., Johnson, A., Matsuda, K., Sadato, N., & Hallett, M. (2007). The role of the human ventral premotor cortex in counting successive stimuli. *Experimental brain research*, *178*(3), 339–350. <https://doi.org/10.1007/s00221-006-0736-8>

Kaufmann, L., Koppelstaetter, F., Delazer, M., Siedentopf, C., Rhomberg, P., Golaszewski, S., Felber, S., & Ischebeck, A. (2005). Neural correlates of distance and congruity effects in a numerical Stroop task: an event-related fMRI study. *NeuroImage*, *25*(3), 888–898. <https://doi.org/10.1016/j.neuroimage.2004.12.041>

Kaufmann, L., Vogel, S. E., Wood, G., Kremser, C., Schocke, M., Zimmerhackl, L. B., & Koten, J. W. (2008). A developmental fMRI study of nonsymbolic numerical and spatial processing. *Cortex; a journal devoted to the study of the nervous system and behavior*, *44*(4), 376–385. <https://doi.org/10.1016/j.cortex.2007.08.003>

Kawashima, R., Taira, M., Okita, K., Inoue, K., Tajima, N., Yoshida, H., Sasaki, T., Sugiura, M., Watanabe, J., & Fukuda, H. (2004). A functional MRI study of simple arithmetic--a comparison between children and adults. *Brain research. Cognitive brain research*, *18*(3), 227–233. <https://doi.org/10.1016/j.cogbrainres.2003.10.009>

Keller, K., & Menon, V. (2009). Gender differences in the functional and structural neuroanatomy of mathematical cognition. *NeuroImage*, *47*(1), 342–352. <https://doi.org/10.1016/j.neuroimage.2009.04.042>

Kong, J., Wang, C., Kwong, K., Vangel, M., Chua, E., & Gollub, R. (2005). The neural substrate of arithmetic operations and procedure complexity. *Brain research. Cognitive brain research*, *22*(3), 397–405. <https://doi.org/10.1016/j.cogbrainres.2004.09.011>

Krueger, F., Spampinato, M. V., Pardini, M., Pajevic, S., Wood, J. N., Weiss, G. H., Landgraf, S., & Grafman, J. (2008). Integral calculus problem solving: an fMRI investigation. *Neuroreport*, *19*(11), 1095–1099. <https://doi.org/10.1097/WNR.0b013e328303fd85>

Kuo, B. C., Yeh, Y. Y., Chen, D. Y., Liang, K. C., & Chen, J. H. (2008). The capacity constraint in the prefrontal and parietal regions for coordinating dual arithmetic tasks. *Brain research*, *1199*, 100–110. <https://doi.org/10.1016/j.brainres.2007.12.070>

Landrø, N. I., Rund, B. R., Lund, A., Sundet, K., Mjellem, N., Asbjørnsen, A., Thomsen, T., Ersland, L., Lundervold, A., Smievoll, A. I., Egeland, J., Stordal, K., Roness, A., Sundberg, H., & Hugdahl, K. (2001). Honig's model of working memory and brain activation: an fMRI study. *Neuroreport*, *12*(18), 4047–4054. <https://doi.org/10.1097/00001756-200112210-00038>

Le Clec'H, G., Dehaene, S., Cohen, L., Mehler, J., Dupoux, E., Poline, J. B., Lehéricy, S., van de Moortele, P. F., & Le Bihan, D. (2000). Distinct cortical areas for names of numbers and body parts independent of language and input modality. *NeuroImage*, *12*(4), 381–391. <https://doi.org/10.1006/nimg.2000.0627>

Lee K. M. (2000). Cortical areas differentially involved in multiplication and subtraction: a functional magnetic resonance imaging study and correlation with a case of selective acalculia. *Annals of neurology*, *48*(4), 657–661.

Leroux, G., Spiess, J., Zago, L., Rossi, S., Lubin, A., Turbelin, M. R., Mazoyer, B., Tzourio-Mazoyer, N., Houdé, O., & Joliot, M. (2009). Adult brains don't fully overcome biases that lead to incorrect performance during cognitive development: an fMRI study in young adults completing a Piaget-like task. *Developmental science*, *12*(2), 326–338. <https://doi.org/10.1111/j.1467-7687.2008.00785.x>

Liu, X., Wang, H., Corbly, C. R., Zhang, J., & Joseph, J. E. (2006). The involvement of the inferior parietal cortex in the numerical Stroop effect and the distance effect in a two-digit number comparison task. *Journal of cognitive neuroscience*, *18*(9), 1518–1530. <https://doi.org/10.1162/jocn.2006.18.9.1518>

Lyons, I. M., & Beilock, S. L. (2013). Ordinality and the nature of symbolic numbers. *The Journal of neuroscience : the official journal of the Society for Neuroscience*, *33*(43), 17052–17061. <https://doi.org/10.1523/JNEUROSCI.1775-13.2013>

Menon, V., Mackenzie, K., Rivera, S. M., & Reiss, A. L. (2002). Prefrontal cortex involvement in processing incorrect arithmetic equations: evidence from event-related fMRI. *Human brain mapping*, *16*(2), 119–130. <https://doi.org/10.1002/hbm.10035>

Menon, V., Rivera, S. M., White, C. D., Glover, G. H., & Reiss, A. L. (2000). Dissociating prefrontal and parietal cortex activation during arithmetic processing. *NeuroImage*, *12*(4), 357–365. <https://doi.org/10.1006/nimg.2000.0613>

Molko, N., Cachia, A., Rivière, D., Mangin, J. F., Bruandet, M., Le Bihan, D., Cohen, L., & Dehaene, S. (2003). Functional and structural alterations of the intraparietal sulcus in a developmental dyscalculia of genetic origin. *Neuron*, *40*(4), 847–858. <https://doi.org/10.1016/s0896-6273(03)00670-6>

Ogata, Y., Horaguchi, T., Watanabe, N., & Yamamoto, M. (2011). Comparison of the choice effect and the distance effect in a number-comparison task by FMRI. *PloS one*, *6*(6), e21716. <https://doi.org/10.1371/journal.pone.0021716>

Olm, C. A., McMillan, C. T., Spotorno, N., Clark, R., & Grossman, M. (2014). The relative contributions of frontal and parietal cortex for generalized quantifier comprehension. *Frontiers in human neuroscience*, *8*, 610. <https://doi.org/10.3389/fnhum.2014.00610>

Pesenti, M., Thioux, M., Seron, X., & De Volder, A. (2000). Neuroanatomical substrates of arabic number processing, numerical comparison, and simple addition: a PET study. *Journal of cognitive neuroscience*, *12*(3), 461–479. <https://doi.org/10.1162/089892900562273>

Piazza, M., Izard, V., Pinel, P., Le Bihan, D., & Dehaene, S. (2004). Tuning curves for approximate numerosity in the human intraparietal sulcus. *Neuron*, *44*(3), 547–555. <https://doi.org/10.1016/j.neuron.2004.10.014>

Piazza, M., Mechelli, A., Butterworth, B., & Price, C. J. (2002). Are subitizing and counting implemented as separate or functionally overlapping processes?. *NeuroImage*, *15*(2), 435–446. <https://doi.org/10.1006/nimg.2001.0980>

Piazza, M., Mechelli, A., Price, C. J., & Butterworth, B. (2006). Exact and approximate judgements of visual and auditory numerosity: an fMRI study. *Brain research*, *1106*(1), 177–188. <https://doi.org/10.1016/j.brainres.2006.05.104>

Piazza, M., Pinel, P., Le Bihan, D., & Dehaene, S. (2007). A magnitude code common to numerosities and number symbols in human intraparietal cortex. *Neuron*, *53*(2), 293–305. <https://doi.org/10.1016/j.neuron.2006.11.022>

Pinel, P., Dehaene, S., Rivière, D., & LeBihan, D. (2001). Modulation of parietal activation by semantic distance in a number comparison task. *NeuroImage*, *14*(5), 1013–1026. <https://doi.org/10.1006/nimg.2001.0913>

Pinel, P., Le Clec'H, G., van de Moortele, P. F., Naccache, L., Le Bihan, D., & Dehaene, S. (1999). Event-related fMRI analysis of the cerebral circuit for number comparison. *Neuroreport*, *10*(7), 1473–1479. <https://doi.org/10.1097/00001756-199905140-00015>

Pinel, P., Piazza, M., Le Bihan, D., & Dehaene, S. (2004). Distributed and overlapping cerebral representations of number, size, and luminance during comparative judgments. *Neuron*, *41*(6), 983–993. <https://doi.org/10.1016/s0896-6273(04)00107-2>

Prabhakaran, V., Rypma, B., & Gabrieli, J. D. (2001). Neural substrates of mathematical reasoning: a functional magnetic resonance imaging study of neocortical activation during performance of the necessary arithmetic operations test. *Neuropsychology*, *15*(1), 115–127. <https://doi.org/10.1037//0894-4105.15.1.115>

Prado, J., Lu, J., Liu, L., Dong, Q., Zhou, X., & Booth, J. R. (2013). The neural bases of the multiplication problem-size effect across countries. *Frontiers in human neuroscience*, *7*, 189. <https://doi.org/10.3389/fnhum.2013.00189>

Rickard, T. C., Romero, S. G., Basso, G., Wharton, C., Flitman, S., & Grafman, J. (2000). The calculating brain: an fMRI study. *Neuropsychologia*, *38*(3), 325–335. <https://doi.org/10.1016/s0028-3932(99)00068-8>

Roggeman, C., Santens, S., Fias, W., & Verguts, T. (2011). Stages of nonsymbolic number processing in occipitoparietal cortex disentangled by fMRI adaptation. *The Journal of neuroscience : the official journal of the Society for Neuroscience*, *31*(19), 7168–7173. <https://doi.org/10.1523/JNEUROSCI.4503-10.2011>

Rosenberg-Lee, M., Chang, T. T., Young, C. B., Wu, S., & Menon, V. (2011). Functional dissociations between four basic arithmetic operations in the human posterior parietal cortex: a cytoarchitectonic mapping study. *Neuropsychologia*, *49*(9), 2592–2608. <https://doi.org/10.1016/j.neuropsychologia.2011.04.035>

Sahan, M. I., Majerus, S., Andres, M., & Fias, W. (2019). Functionally distinct contributions of parietal cortex to a numerical landmark task: An fMRI study. *Cortex; a journal devoted to the study of the nervous system and behavior*, *114*, 28–40. <https://doi.org/10.1016/j.cortex.2018.11.005>

Sammer, G., Blecker, C., Gebhardt, H., Bischoff, M., Stark, R., Morgen, K., & Vaitl, D. (2007). Relationship between regional hemodynamic activity and simultaneously recorded EEG-theta associated with mental arithmetic-induced workload. *Human brain mapping*, *28*(8), 793–803. <https://doi.org/10.1002/hbm.20309>

Santens, S., Roggeman, C., Fias, W., & Verguts, T. (2010). Number processing pathways in human parietal cortex. *Cerebral cortex (New York, N.Y. : 1991)*, *20*(1), 77–88. <https://doi.org/10.1093/cercor/bhp080>

Simon, O., Mangin, J. F., Cohen, L., Le Bihan, D., & Dehaene, S. (2002). Topographical layout of hand, eye, calculation, and language-related areas in the human parietal lobe. *Neuron*, *33*(3), 475–487. <https://doi.org/10.1016/s0896-6273(02)00575-5>

Stanescu-Cosson, R., Pinel, P., van De Moortele, P. F., Le Bihan, D., Cohen, L., & Dehaene, S. (2000). Understanding dissociations in dyscalculia: a brain imaging study of the impact of number size on the cerebral networks for exact and approximate calculation. *Brain : a journal of neurology*, *123 ( Pt 11)*, 2240–2255. <https://doi.org/10.1093/brain/123.11.2240>

Tan, H. Y., Chen, Q., Goldberg, T. E., Mattay, V. S., Meyer-Lindenberg, A., Weinberger, D. R., & Callicott, J. H. (2007). Catechol-O-methyltransferase Val158Met modulation of prefrontal-parietal-striatal brain systems during arithmetic and temporal transformations in working memory. *The Journal of neuroscience : the official journal of the Society for Neuroscience*, *27*(49), 13393–13401. <https://doi.org/10.1523/JNEUROSCI.4041-07.2007>

Tenison, C., Fincham, J. M., & Anderson, J. R. (2014). Detecting math problem solving strategies: an investigation into the use of retrospective self-reports, latency and fMRI data. *Neuropsychologia*, *54*, 41–52. <https://doi.org/10.1016/j.neuropsychologia.2013.12.011>

Tiberghien, K., De Smedt, B., Fias, W., & Lyons, I. M. (2019). Distinguishing between cognitive explanations of the problem size effect in mental arithmetic via representational similarity analysis of fMRI data. *Neuropsychologia*, *132*, 107120. <https://doi.org/10.1016/j.neuropsychologia.2019.107120>

van der Ven, F., Takashima, A., Segers, E., Fernández, G., & Verhoeven, L. (2016). Non-symbolic and symbolic notations in simple arithmetic differentially involve intraparietal sulcus and angular gyrus activity. *Brain research*, *1643*, 91–102. <https://doi.org/10.1016/j.brainres.2016.04.050>

Venkatraman, V., Ansari, D., & Chee, M. W. (2005). Neural correlates of symbolic and non-symbolic arithmetic. *Neuropsychologia*, *43*(5), 744–753. <https://doi.org/10.1016/j.neuropsychologia.2004.08.005>

Venkatraman, V., Siong, S. C., Chee, M. W., & Ansari, D. (2006). Effect of language switching on arithmetic: a bilingual FMRI study. *Journal of cognitive neuroscience*, *18*(1), 64–74. <https://doi.org/10.1162/089892906775250030>

Vogel, S. E., Grabner, R. H., Schneider, M., Siegler, R. S., & Ansari, D. (2013). Overlapping and distinct brain regions involved in estimating the spatial position of numerical and non-numerical magnitudes: an fMRI study. *Neuropsychologia*, *51*(5), 979–989. <https://doi.org/10.1016/j.neuropsychologia.2013.02.001>

Vogel, S.E., Goffin, C., Bohnenberger, J., Koschutnig, K., Reishofer, G., Grabner, R.H., & Ansari, D. (2017). The left intraparietal sulcus adapts to symbolic number in both the visual and auditory modalities: Evidence from fMRI. *NeuroImage, 153*, 16-27.

Wood, G., Nuerk, H. C., Moeller, K., Geppert, B., Schnitker, R., Weber, J., & Willmes, K. (2008). All for one but not one for all: how multiple number representations are recruited in one numerical task. *Brain research*, *1187*, 154–166. <https://doi.org/10.1016/j.brainres.2007.09.094>

Yang, Y., Zhong, N., Friston, K., Imamura, K., Lu, S., Li, M., Zhou, H., Wang, H., Li, K., & Hu, B. (2017). The functional architectures of addition and subtraction: Network discovery using fMRI and DCM. *Human brain mapping*, *38*(6), 3210–3225. <https://doi.org/10.1002/hbm.23585>

Yi-Rong, N., Si-Yun, S., Zhou-Yi, G., Si-Run, L., Yun, B., Song-Hao, L., & Chan, W. Y. (2011). Dissociated brain organization for two-digit addition and subtraction: an fMRI investigation. *Brain research bulletin*, *86*(5-6), 395–402. <https://doi.org/10.1016/j.brainresbull.2011.08.016>

Yusoff, A., Sok Bee, N., Xin Ling, T., & Abd. Hamid, A. (2014). Investigating Brain Activation and Neural Efficacy During Simple Arithmetic Addition Task in Quiet and in Noise: An fMRI Study*. Jurnal Sains Kesihatan Malaysia (Malaysian Journal of Health Sciences), 12*(1). Retrieved from <http://ejournal.ukm.my/jskm/article/view/6991/2874>

Zago, L., Pesenti, M., Mellet, E., Crivello, F., Mazoyer, B., & Tzourio-Mazoyer, N. (2001). Neural correlates of simple and complex mental calculation. *NeuroImage*, *13*(2), 314–327. <https://doi.org/10.1006/nimg.2000.0697>

Zago, L., Petit, L., Turbelin, M. R., Andersson, F., Vigneau, M., & Tzourio-Mazoyer, N. (2008). How verbal and spatial manipulation networks contribute to calculation: an fMRI study. *Neuropsychologia*, *46*(9), 2403–2414. <https://doi.org/10.1016/j.neuropsychologia.2008.03.001>

Zhou, X., Chen, C., Zang, Y., Dong, Q., Chen, C., Qiao, S., & Gong, Q. (2007). Dissociated brain organization for single-digit addition and multiplication. *NeuroImage*, *35*(2), 871–880. <https://doi.org/10.1016/j.neuroimage.2006.12.017>

**Supplementary Information C**

**Checklist for neuroimaging meta-analysis (Muller et al. 2018)**

| The research question is specifically defined | **YES** and it includes the following contrasts:  Aim 1:   - spatial processing > control conditions - temporal processing > control conditions - numerical processing > control conditions   Aim 2:   - (spatial processing > control conditions) > (temporal processing > control conditions) - (spatial processing > control conditions) < (temporal processing > control conditions) - (numerical processing > control conditions) > (spatial processing > control conditions) - (numerical processing > control conditions) < (spatial processing > control conditions) - (numerical processing > control conditions) > (temporal processing > control conditions) - (numerical processing > control conditions) < (temporal processing > control conditions)   Aim 3:   - (numerical processing > control conditions) ∩ (spatial processing > control conditions) - (numerical processing > control conditions) ∩ (temporal processing > control conditions) - (space processing > control conditions) ∩ (temporal processing > control conditions) - (numerical processing > control conditions) ∩ (spatial processing > control conditions) ∩ (temporal processing > control conditions) |
| --- | --- |
| The literature search was systematic | **YES (PRISMA guidelines used)**, it includes the following keywords in the following databases:  **Keywords**:  For Space processing:  ((((((((mental rotation OR topographic OR (spatial AND (attention OR processing OR navigation OR memory) NOT time)) AND ((functional AND magnetic AND resonance AND imaging) OR fMRI)) NOT (structural OR DWI OR DTI OR diffusion OR machine learning OR multivariate OR multivoxel)) NOT (EEG OR transcranial OR stimulation OR tDCS))NOT (dementia OR deficit OR disorder OR pathology OR disease OR neglect OR cardia* OR Parkinson OR Alzheimer OR addiction OR drug OR psychiatric OR schizoph* OR psychosis OR neurologic OR injury OR stroke OR cannabis)) NOT (aging OR elderly OR children OR childhood)) NOT (mouse OR mice OR rat OR animal OR monkey)) NOT (meta-analysis[title] OR review[title] OR review[Publication Type] OR meta-analysis[Publication Type] OR Case Reports[Publication Type]))  For Time processing:  (((((((((time OR temporal) AND (duration OR processing OR perception) NOT (space OR spatial))) AND ((functional AND magnetic AND resonance AND imaging) OR fMRI)) NOT (structural OR DWI OR DTI OR diffusion OR machine learning OR multivariate OR multivoxel OR PET OR positron OR connectivity OR convolutional OR “deep learning”)) NOT (EEG OR transcranial OR stimulation OR tDCS)) NOT (dementia OR deficit OR disorder OR pathology OR disease OR neglect OR cardia* OR Parkinson OR Alzheimer OR addiction OR drug OR psychiatric OR schizoph* OR psychosis OR neurologic OR injury OR stroke OR cannabis)) NOT (aging OR elderly OR children OR childhood)) NOT (mouse OR mice OR rat OR animal OR monkey)) NOT (meta-analysis[title] OR review[title] OR review[Publication Type] OR meta-analysis[Publication Type] OR Case Reports[Publication Type]))  For Numbers processing:  ((((((((number OR numerosity OR calculation OR multiplication OR subtraction OR addition OR division OR ‘’ Numbers comparison’’ OR ‘’arithmetical problems’’ OR ‘’arithmetical task’’) AND (fMRI OR “functional magnetic resonance” OR PET OR “Positron emission tomography”)) NOT (structural OR DWI OR DTI OR diffusion OR machine learning OR multivariate OR multivoxel)) NOT (EEG OR transcranial OR stimulation OR tDCS OR MRI)) NOT (dementia OR deficit OR disorder OR pathology OR disease OR neglect OR cardia OR Parkinson OR Alzheimer OR addiction OR drug OR psychiatric OR schizoph OR psychosis OR neurologic OR injury OR stroke OR cannabis OR cancer OR radiologist OR clinical OR seizures OR lesions OR acalculia OR visual OR time OR space OR theraphy OR dyslexia)) NOT (aging OR elderly OR children OR childhood OR patients)) NOT (mouse OR mice OR rat OR animal OR monkey OR dog OR pets)) NOT (meta-analysis[title] OR review[title] OR review[Publication Type] OR meta-analysis[Publication Type] OR Case Reports[Publication Type]))  **Databases**: PubMed, MEDLINE, Google Scholar, “related article” function in PubMed, reference within the selected literature. |
| Detailed inclusion and exclusion criteria are included | **YES**, and reason for non-standard criterion was:  Standard criteria applied: only whole brain experiment included; only studies reporting results in a standardized coordinate space were included.  Non standard criteria applied:   - studies that used fMRI or PET 🡪 criterion decided in order to maximize the power of the meta-analysis and in order to exclude studies with structural MRI; - studies analyzing the data using univariate approach that revealed localized increased activation were included 🡪 criterion decided to exclude papers that analyzed data using machine learning, whose results have a slightly different meaning; and to exclude papers using functional connectivity techniques, as we are not interested in connectivity; - studies with sample size of at least 5 participants (per group) were included 🡪 criterion decided according with previous coordinate based meta-analysis in order to reduce the likelihood to include studies presenting false positives; - studies were included only if they are performed on healthy individuals 🡪 criterion decided in accordance with the research question (to investigate the neural basis of normal spatial or temporal processing); - only studies reporting the contrast space or time or numbers (symbolic, non-symbolic and arithmetic elaboration) > control condition were included 🡪 criterion decided to increase the specificity of the meta-analysis. - studies that did not focus on isolating specific brain regions’ activations related to a given numerical, spatial, or temporal function (e.g., parahippocampal gyrus and posterior cingulate cortex) 🡪 criterion decided to highlight the activations linked to numbers, space and time processing rather than the activations associated with a specific spatial function (e.g., navigation or long-term memory) |
| Sample overlap was taken into account | **YES**, using the following method:  For each paper, only the contrast that most strongly reflects the process that the meta-analysis aims to investigate has been selected. In few cases, more contrasts were selected for a single paper: in all these cases, the authors run the analysis on two independent samples, and this is clearly stated in the paper. |
| All experiments use the same search coverage (state how brain coverage is assessed and how small volume corrections and conjunctions are taken into account) | **YES**, the search coverage is the following:  Whole brain.  If a study reported whole brain + ROI analysis, the whole brain analysis only has been included in the meta-analysis; if a study reported the ROI analysis only, the study was excluded from the meta-analysis in accordance with the inclusion/exclusion criteria.  We found no paper that applied small volume correction or hidden ROI. Papers reporting only conjunction analysis have been excluded. |
| Studies are converted to a common reference space | **YES**, using the following conversion:  Tailarach coordinates were reported into MNI space using a linear transformation as implemented in GingerALE 3.0.2 software. |
| Data extraction have been conducted by two investigators (ideal case) or double checked by the same investigator (state how double checking was performed) | **YES**, the following authors:  CS, VF and FA checked inclusion criteria;  CS, VF and FA extracted coordinates;  CS, VF and FA extracted other info: Number of subjects included, Type of task (cognitive function involved); task (specific task); contrast performed; coordinate system; coordinate localization; associated statistic (t value, z score); p value criteria (corrected, uncorrected);  GC and MW double-checked the following data: extracted coordinates randomly and when discordant coordinates were extracted by CS, VF and FA. |
| The paper includes a table with at least the references, basic study description (e.g. for fMRI task: stimuli), contrasts and basic sample descriptions (e.g. size, mean age and gender distribution, etc) of the included studies, source of information, reference space | **YES** and also the following data:  Study reference; Number of subjects included; task (specific task); contrast performed; coordinate system; coordinate localization; associated statistic (t value, z score); p value criteria (corrected, uncorrected);  **Please note that in this case the table is not enclosed within the paper but it is available within the supplementary information as an excel database.** |
| The study protocol was previously registered and all analyses planned beforehand, including the methods and parameters used for inference, correction for multiple testing, etc. | **NO**:   1. the meta-analysis was not registered before starting the search. Indeed, according with PROSPERO, systematic review and meta-analysis should be registered only if they are relevant to health and social care, which is not the case (https://www.crd.york.ac.uk/prospero/); 2. We declared that we planned all the analysis before starting the literature search and that we did not run any non-planned or non-prespecified analysis; 3. The meta-analysis used the default methods and parameters of the software with the following exceptions: none. |
| The meta-analysis includes diagnostics | NO. |
